# New Insights on Endophytic *Microbacterium*-Assisted Blast Disease Suppression and Growth Promotion in Rice: Revelation by Polyphasic Functional Characterization and Transcriptomics

**DOI:** 10.3390/microorganisms11020362

**Published:** 2023-01-31

**Authors:** Asharani Patel, Kuleshwar Prasad Sahu, Sahil Mehta, Mohammed Javed, Alexander Balamurugan, Mushineni Ashajyothi, Neelam Sheoran, Prakash Ganesan, Aditi Kundu, Subbaiyan Gopalakrishnan, Robin Gogoi, Aundy Kumar

**Affiliations:** Division of Plant Pathology, ICAR-Indian Agricultural Research Institute, New Delhi 110012, India

**Keywords:** blast disease, *Magnaporthe*, *Microbacterium testaceum*, probiotic features, transcriptome assembly, DEGs

## Abstract

Plant growth-promoting endophytic microbes have drawn the attention of researchers owing to their ability to confer fitness benefits in many plant species. Here, we report agriculturally beneficial traits of rice-leaf-adapted endophytic *Microbacterium testaceum*. Our polyphasic taxonomic investigations revealed its identity as *M. testaceum*. The bacterium displayed typical endophytism in rice leaves, indicated by the green fluorescence of GFP-tagged *M. testaceum in* confocal laser scanning microscopy. Furthermore, the bacterium showed mineral solubilization and production of IAA, ammonia, and hydrolytic enzymes. Tobacco leaf infiltration assay confirmed its non-pathogenic nature on plants. The bacterium showed antifungal activity on *Magnaporthe oryzae*, as exemplified by secreted and volatile organic metabolome-mediated mycelial growth inhibition. GC-MS analysis of the volatilome of *M. testaceum* indicated the abundance of antimicrobial compounds. Bacterization of rice seedlings showed phenotypic traits of MAMP-triggered immunity (MTI), over-expression of *OsNPR1* and *OsCERK*, and the consequent blast suppressive activity. Strikingly, *M. testaceum* induced the transcriptional tradeoff between physiological growth and host defense pathways as indicated by up- and downregulated DEGs. Coupled with its plant probiotic features and the defense elicitation activity, the present study paves the way for developing *Microbacterium testaceum-*mediated bioformulation for sustainably managing rice blast disease.

## 1. Introduction

Since time immemorial, rice has been one of the most critical staples contributing to the increasing human population’s food security and dietary requirement. The UN World Food Program estimated acute food insecurity globally—an increase of 82% over the pre-COVID-19 prediction [1,2]. The burgeoning human population and the consequent shrinking agricultural habitats, in conjunction with climate change risks and biotic stress, further compound the global food security challenges [3,4,5]. Among the biotic stresses, the rice blast disease caused by the fungus *Magnaporthe oryzae* (syn: *Pyricularia oryzae*) (Herbert) Barr poses a threat to 85 rice-growing nations [6,7,8,9]. Besides rice, M. *oryzae is* known to infect other economic monocots such as wheat, oats, barley, finger millet, and pearl millet, posing a challenge to food and nutritional security [10]. The preventive blast management strategies include the deployment of resistant cultivars and the application of fungicides. However, the durability of these options is questionable owing to the emergence of new pathotypes of the pathogen and unacceptable toxicity residues [11].

To meet future rice production challenges, there is an urgent need to develop strategies acceptable to all rice farming stakeholders. The plant microbiome, the phytobiome, presents a novel opportunity to harness pathogen-competing and crop-cooperating microbiota [12]. Rice is a tropical plant colonized by diverse microorganisms such as epiphytes and endophytes [13,14]. Endophytic bacteria-based bioinoculants are increasingly being used in agriculture owing to metabolic capabilities such as the secretion of antimicrobial metabolites and the elicitation of host defense [15,16]. For example, endophytic *Bacillus subtilis* var. *amyloliquefaciens* strains EPB9, EPB10, EPCO29, and EPCO78 significantly suppressed bacterial leaf blight [17]. Likewise, *Bacillus amyloliquefaciens* strains EPB9, EPB10, EPCO29, and EPCO78 significantly suppressed bacterial leaf blight [17]. In another case, endophytic *B. subtilis* E1R-j suppressed the fungal pathogen *Gaeumannomyces graminis* var. *tritici* by its antifungal activity [18]. *Pseudomonas putida* BP25, an endophyte of black pepper, emits a broad-spectrum antifungal bacterial volatile compound belonging to pyrazines [19]. Endophytic *P. putida* BP25 showed bacterial volatile-mediated antifungal activity against rice blast fungus *Magnaporthe oryzae* 1637 and also induced the rice defense-related gene PAD4 [20]. In another report, rice phyllosphere-associated bacteria *Stenotrophomonas* sp. and *Microbacterium oleivorans* were found to suppress blast disease severity by more than 60% by triggering the expression of defense genes such as *OsCEBiP*, *OsCERK1*, *OsEDS1*, and *OsPAD4* [21].

Similarly, *Microbacterium testaceum*, *Enterobacter sacchari*, *Pantoea dispersa*, *Pantoea ananatis*, *Pantoea vagans*, *Pseudomonas oryzihabitans*, *Rhizobium* sp., and *Sphingomonas* sp. exhibited suppressive effect against rice blast pathogen and also triggered host defense mechanism [12]. Kumar and colleagues in the year 2021 reported the presence of core and satellite endophytic microbiome on rice phyllosphere comprising *Alcaligenes*, *Acidovorax*, *Bacillus*, *Comamonas*, *Pseudomonas*, *Pantoea*, *Chryseobacterium*, *Microbacterium*, etc. [22]. Rice endophytic bacteria *Chryseobacterium endophyticum* was found to suppress rice blast fungus by releasing several volatile compounds, which also induced several rice plant-associated defense genes (*OsCERK*, *OsCEBiP*, *OsNPR1.3*, *OsPAD4*, and *OsFMO1*) [23]. However, the mechanisms involved in the induction of plant defense mechanisms upon treatment with endophytes are poorly understood.

Within these endophytic bacteria, the genus *Microbacterium* has yet to be explored much in the literature. With this background, we attempted to explore the biostimulant potential of the rice-endophytic bacterium *Microbacterium testaceum* Os-Enb-ALMB2. After isolation and identification, an in-depth taxonomic and functional characterization was conducted using multi-omics tools. We further established the endophytism of the bacterium in rice plants using the gfp-reporter gene. Lastly, both in vitro and in vivo antifungal activity against rice blast pathogen and defense elicitation against blast disease were evaluated using several markers of plant defense-related genes through qRT-PCR. The results on the elicitation of defense genes prompted us to perform transcriptomics on the bacteria-treated rice seedlings. The results of our study are expected to contribute to sustainable rice blast disease management by reducing the use of agrochemicals in food production.

## 2. Materials and Methods

### 2.1. Bacterial Strain and Identity Confirmation

The bacterial strain *Os*Enb-ALM-B2 used in this study was initially isolated from the leaf tissue of rice (*Oryza sativa* L.) cultivar Pusa Basmati-6 (PB6) planted in Almora, Uttarakhand (29.58916667 N 79.64388889 E) [22,23]. The 16S rRNA gene sequencing was used to identify the strain as *Microbacterium testaceum*, and the same was submitted to NCBI GenBank with the accession number MN889362 (https://www.ncbi.nlm.nih.gov; accessed on 31 October 2022). For routine work, the culture (stored as a glycerol stock) was streaked on Nutrient agar media (NA, gL−1 Peptone 5.0; Beef extract 3.0; NaCl 5.0; Agar 15.0; pH 7.0 ± 0.2) and incubated at 28 °C for 48 h after being retrieved from −80 °C.

Using the negative staining procedure, the bacterium *Microbacterium testaceum Os*Enb-ALM-B2 (hereafter *M. testaceum*B2) was observed in the transmission electron microscope (TEM) at magnifications more than 60K× [24]. After being cultivated in a NA medium for 24 h at 28 °C, Biolog-based phenotypic fingerprinting was performed to confirm the species identity of the isolate. The bacterial isolate was suspended in Inoculation Fluid-A (IF-A) GN/GP base inoculating fluid at a density equivalent to 95–98% transmittance, as per the manufacturer’s instructions (Biolog, Hayward, CA, USA). The prepared bacterial cell suspension was injected (100 µL/well) into a GEN-III plate and incubated for 18–24 h at 33 °C before being scanned at A570nm with a MicroPlate reader [25]. The species identification was validated by comparison with the Biolog database (Biolog, Hayward, CA, USA).

### 2.2. Multigene-Sequencing-Based Identification

Further, multigene sequencing was used to confirm the bacterial species identity using eight house-keeping genes specific to *Microbacterium testaceum*, such as *rpoC* (DNA-directed RNA polymerase subunit beta′), *pyk* (Pyruvate kinase), *gyrB* (DNA gyrase subunit B), *tyrS* (Tyrosine—tRNA ligase), *infB* (Eukaryotic initiation factor IF-2), *fumC* (Fumaratehydratase), *metG* (Methionine- tRNA ligase), and *tyrS* (Tyrosine- tRNA ligase) (Table S1).

The total genomic DNA was isolated using a modified CTAB method [12,21]. The PCR was performed in a thermal cycler with the reaction mixture and conditions described in Tables S2 and S3, respectively. The amplicons were bidirectionally sequenced using the Sanger sequencing platform, curated, and analyzed using the BLAST algorithm available in NCBI GenBank. Multi-gene sequence-based phylogenetic analysis was also performed using the Maximum Likelihood approach and Hasegawa–Kishino–Yano model with 1000-bootstrap support using the MegaX program [26,27,28].

### 2.3. Antagonistic Activity of Microbacterium testaceum B2 on Magnaporthe oryzae In Vitro

After establishing the identity and multigene analysis, the antagonistic activity of *M. testaceum* B2 on *Magnaporthe oryzae* 1637, a dual culture confrontation assay of bacteria-secreted metabolite, was performed on the Petri plates (40.0 mm). First, the fungal mycelial disc (0.5 cm diameter) was inoculated at one corner of the plate using a modified PDA-TSA medium [(Potato dextrose agar (SRL, Mumbai, India)-Tryptic soy agar (HiMedia Laboratories, Mumbai, India)]. Then, the bacterial inoculum was streaked parallel to the fungal mycelial disc on the agar surface.

Using the Sheoran et al. [19] methodology, the antifungal activity of volatile organic compounds emitted by *M. testaceum* B2 was investigated against *M. oryzae* 1637. A mycelial disc was inserted in the center of a PDA plate. Similarly, 20 µL of 10^8^ CFU mL^−1^ mid-log phase bacterial cultures were placed on a Tryptic soy agar plate (HiMedia Laboratories, Mumbai, India) on another Petri plate. The lids of both Petri plates were removed, and the inoculated plate was placed face-to-face and secured with parafilm, Petri seal tape, and clean film so that the volatile could not escape. For both assays, 3 replications of the plates were incubated at 28 °C for 5–7 days. Petri plates with no bacterial inoculums served as a control. Later, the colony diameter was measured, and mycelia % growth inhibition of the pathogen was determined using the following formula.

Inhibition (%) = C − T/C × 100

where C = Colony diameter in control, and T = Colony diameter in treatment.

### 2.4. Chemo-Profiling of Bacterial Volatile Organic Compounds with Solvent Extraction (SE) Method

The volatiles produced by *M. testaceum* B2 could completely inhibit the fungal growth in vitro, which further led to the investigation volatile profile of the bacterial isolate. For this investigation, bacterial volatiles and organic compounds were recovered using the solvent extraction method from a 72-h-old bacterial culture in the HPLC grade Hexane [29]. In addition, gas Chromatography–Mass Spectroscopic analysis was also performed on the solvent Hexane, which was dissolved with bacterial organic compounds (GC-MS).

For GC-MS analysis, an HP-5MS column (60 m 0.25 mm;/0.25 m; Agilent Technologies, Inc., Santa Clara, CA, USA) was used, which was immediately linked to a triple-axis mass spectrometer (Thermo Fisher, Waltham, MA, USA). The injection volume was 1.0 µL with flow mode in split control. The flow rate of the Helium (He) gas carrier was fixed at 1.0 mL min^−1^, where the He gas (He-High purity, Amit-Gas, New Delhi, India) was used at a head pressure of 10 psi. The oven temperature condition and MS acquisition settings were adapted from Kumar et al. [23]. Chemical compounds were identified by comparing mass spectra. For establishing the fundamental components, the National Institute of Standards and Technologies (NIST) Mass Spectra Library was used as a reference.

### 2.5. Testing the Ability of Microbacterium testaceum B2 to Induce a Hypersensitive Reaction (HR) on Nicotiana tabacum

Tobacco (*Nicotiana tabacum*.) plants were cultivated in greenhouse conditions. The experiment included 2-or 3-month-old plants with fully developed 7–8 leaves. The plants were given pre-inoculation conditioning in a greenhouse with a temperature of 20 °C, 50–60% relative humidity, and a 12-h light/dark cycle for two successive days. All of the trials were conducted with fully developed leaves that were attached to the plants. First, a bacterial suspension of 1 × 10^8^ CFU mL^−1^ (1.0 OD A600nm) was prepared on sterile distilled water. Then, using a sterile hypodermal syringe, it was infiltrated on potted *N. tabacum* eaves and incubated at 25–30 °C under glasshouse conditions (12 h of dark/light photoperiods).

Negative control of leaves infiltrated with sterile distilled water, whereas a positive control of leaves infiltrated with plant pathogenic bacterium, *Ralstonia solanacearum*, was kept during the experiment. At 24 h post-inoculation (hpi), the infiltrating bacteria’s induced responses on the tobacco leaves were recorded [30].

### 2.6. Studies on Endophytic Colonization of Microbacterium testaceum B2 in Rice Plant

The *M. testaceum* B2 strain with intrinsic rifamycin antibiotic resistance was transformed and tagged with a stable green fluorescent protein (GFP) gene. The GFP gene was transferred to the genome and incorporated into a neutral location. To genetically tag the bacterium, we employed the Tn7-based GFP construct ‘pBKminiTn7gfp2Gm10′ in *E. coli* XL1 Blue (Gentamycin 20 g mL^−1^) and a helper plasmid ‘pUXBF13Amp100′ in *E. coli* XL1 Blue (Ampicillin 100 g mL^−1^). The tri-parental-mating approach introduced the GFP gene into these bacteria [19]. The transformants were chosen at 28 °C for 48–72 h on NA plates altered with Rifamycin (50 g mL^−1^) and Gentamycin (20 g mL^−1^).

On freshly NA plates modified with the same antibiotics, putative transformants that appeared on the selection plates were sub-cultured, followed by PCR validation of the insertion on the colonies using a GFP-specific forward primer and reverse primer [16]. Briefly, primer pairs gfp Rt F-5′GGCCGATGCAAAGTGCCGATAAA3′ and gfp Rt R-5′ AGGGCGAAGAATCTCGTGCTTTCA3′ were used in PCR reaction using GoTaq PCR kit (GoTaqBuffer—1X, MgCl_2_—1.5 mM, dNTPs—200 mM, forward/reverse primers—10 pmol each, DMSO—6%, Taq polymerase—1 U; Promega Corporation, Madison, WI, USA) at an initial denaturation at 95 °C for 5 min, 35 cycles of denaturation at 95 °C for 1 min, annealing at 53 °C for 30 s and extension at 72 °C for 30 s followed by a final extension at 72 °C for 5 min. PCR amplicon of 142 bp was visualized in 2% agarose gel (QuantityOne Image Analysis system, BioRad, Hercules, CA, USA). The colonies that yielded positive PCR findings were imaged using a confocal microscope (CLSM -DM6000, Leica Microsystems Wetzlar, Germany) to ensure stable GFP expression. Furthermore, the transformed bacterial strain with stable GFP expression is designated *Microbacterium testaceum* B2R::gfp and preserved as glycerol stock for downstream work.

Internal colonization in the rice was tested using genetically tagged *M. testaceum* B2R::gfp on Pusa Basmati-1. *M. testaceum* B2R::gfp was grown on NA supplemented with the antibiotics reported before. Surface sterilized rice seeds were inoculated for 24 h with a bacterial suspension containing 10^8^ CFU mL^−1^ (1.0 OD at A600nm) and cultured for the next seven days in sterile Petri plates under greenhouse conditions (RH > 90% and temperature 28–30 °C). In addition, untreated seeds were soaked in sterile deionized water that served as a control. Finally, the seedlings were transplanted into small pots filled with sterilized soil and grown in a greenhouse. One-month-old rice plantlets were excised from *M. testaceum* B2R::gfp treated seeds, and thin sections of various plant parts were prepared after being fixed in *p*-formaldehyde (4.0%) for 12 h at 4 °C. Confocal microscope (DM6000, Leica Microsystems Wetzlar, Germany) was used to scan and photograph samples at multiple locations (root, stem, and leaf cross-sections). The images were processed and analyzed to see where the bacteria were present.

A PCR-based assay of the endophytic bacterium *M. testaceum* B2R::gfp was conducted using GFP-specific primers (gfp Rt F-5′GGCCGATGCAAAGTGCCGATAAA3′ and gfp Rt R-5′ AGGGCGAAGAATCTCGTGCTTTCA3′). The treated plant’s root, shoot, and leaves were crushed separately for the tissue colonization study, and 1 mL of water extract from each tissue was centrifuged to obtain the pellet. The resultant pellet was utilized to isolate the complete genomic DNA using the earlier described bacterial DNA isolation procedure. The reaction mixture’s composition and reaction conditions were similar to that of those employed in the selection of GFP transformants. Later, the PCR amplicons were separated in an EtBr-added agarose gel and observed with a UV trans-illuminator (QuantityOne, BioRad, Hercules, CA, USA).

One-month-old PB1 seedlings treated with *M. testaceum* B2R::gfp were surface sterilized in NaClO (1.0%) and ethyl alcohol (70%) before being washed five times in sterile deionized water and utilized after blotting. PBS (Phosphate-Buffered Saline, g L−1: NaCl 8; KCl 0.2; Na_2_HPO_4_ 1.44; KH_2_PO_4_ 0.24; pH 7.4) was used to aseptically grind 1 g tissue from root and leaf samples. The aliquot was serially diluted up to 10^−4^ before being poured plated on NA plates adjusted with rifamycin (50 g mL^−1^) and gentamycin (20 g mL^−1^) and incubated for 48–72 h at 28 °C. Two biological and three technical replications were used in the experiment. In addition, the number of colonies that appeared was counted, and the mean endophytic population was calculated and expressed.

### 2.7. Effect of Microbacterium testaceum B2 Mediated Seed Priming on Rice Seedlings Growth and Development

In the rice cultivars (PB1 and BPT5204), *M. testaceum* B2 was used for biopriming to test the effect on germination, root/shoot length, many roots, and biomass (fresh weight and dry weight). The bacterium was cultured on NA supplemented with the antibiotics mentioned earlier. Surface sterilized rice seeds were soaked for 24 h in decimal dilutions of mid-log phase bacterial suspension with varying cell concentrations, i.e., 10^8^ and 10^7^ cells mL^−1^, and allowed to grow in sterile Petri dishes incubated for seven days under greenhouse conditions (RH > 90% and temperature 28–30 °C). The plant characteristics phenotypes were measured seven days after inoculation in addition to germination. Control seeds were soaked in sterile deionized water. The experiment was repeated twice, with each of the three replications comprising 20 seeds.

### 2.8. Assessing the Probiotic Plant Features of Microbacterium testaceum B2

A variety of plant probiotic properties was explored for *M. testaceum* B2. All studies were conducted in triplicates using a ten μL bacterial suspension in sterile water as the treatment, and 10 μL sterile water without bacteria served as a control. The phosphate solubilization activity was measured using a solid agar medium containing tricalcium phosphate (CaP; 3 gL^−1^), as described by Chen et al. [31]. Using a solid agar medium with an inorganic potassium source Potassium Alumino Silicate, Aleksandrov et al. [32] assessed potassium (K) solubilizing activity was evaluated. In a modified Pikovskaya agar medium containing insoluble zinc (Zn) obtained from ZnO, the solubilization of the mineral Zn was also observed, according to Pikovskaya [33]. The ability to form a siderophore was tested using CAS agar medium as the substrate. The method described by Patten and Glick [34] was used to assess Indole-3-acetic acid (IAA) synthesis using L-tryptophan supplied with DF-salt minimal medium and Salkowski’s reagent. After adding Nessler’s Reagent, as specified by Zhou et al. [35], the ability to emit ammonia was tested in Nutrient Broth.

Under in vitro conditions, *M. testaceum* B2 was also tested for its ability to produce several defense-related enzymes. The bacterium *M. testaceum* B2 was inoculated on starch agar, casein agar, xylan agar, carboxy methyl cellulose (CMC) agar, and pectin agar medium to assess the production of amylase, protease, xylanase, cellulase, and pectinase, respectively, using the agar well diffusion technique [36]. To summarize, 1% of each substrate was used to make substrate media plates in the Nutrient agar medium. Then, wells with a diameter of 5 mm were bored into the solidified media with a cork borer, and the culture suspensions (10 µL of 10^7^ CFU mL^−1^) were added.

The plates were incubated at 28 °C for 48 h, and clear zones surrounding the wells were recorded, with a diameter of 15 mm regarded as a positive result. The enzymes amylase and pectinase were detected by flooding the plates with an iodine–potassium iodide solution for 15 min and then de-staining for 10 min with sodium chloride (1 mol L^−1^). By flooding the plates with Congo Red solution (1.0%) for 15 min and then de-staining with sodium chloride (1 mol L^−1^) for 10 min, cellulase and xylanase enzymes were detected. On casein agar, clear zones around the wells were developed to assess protease production. Inoculating bacteria (5 μL) on nutrient agar supplemented with 1% (*v*/*v*) colloidal chitin and incubated for 1 week; the culture plates were flooded with 1% Congo red, and the development of an orange zone around the colony was marked as positive.

### 2.9. In Planta Evaluation of Microbacterium testaceum B2 for Blast Disease Suppression on Rice

Seeds of blast-susceptible cultivars (PB1 and BPT5204) were bacterized with 10^8^ and 10^7^ CFU mL^−1^ of bacterial suspension (1.0 OD and 0.1 OD at A600nm, respectively) and allowed to germinate for the next 7days. The seedlings were transplanted into small 2-inch pots with sterile farm soil after germination and allowed to grow under greenhouse conditions at 28 ± 2 °C with 85 ± 10% relative humidity (RH) and a 14/12 h light/dark cycle until they attained the 3-leaf stage. *M. oryzae* 1637 was used in the experiment for foliar inoculation after being cultured on rice straw extract sucrose agar (RSESA) medium at 25 °C for seven days. A glass atomizer was used to spray a prophylactic spray of bacterial cell suspension above the foliage 48 h before pathogen inoculation. The conidial suspension (~2 × 10^5^ conidia mL^−1^) was prepared and sprayed over the leaves of 3-week-old seedlings with Tween 20 (0.05%) [11].

Plantlets sprayed with the fungicide tricyclazole (0.1%) were a positive check, while plants not treated with bacteria were a negative control. Inoculated plantlets were incubated in the dark for 24 h at 22 ± 2 °C with 90% RH in a climate-controlled greenhouse. After that, plants were kept under greenhouse conditions with a 12 h dark/light cycle, temperature 22 ± 2 °C, 90% RH, with leaf wetness maintained by spraying water three times a day. Then, 7–10 days after inoculation, the severity of the blast was evaluated using a 0–5 disease rating scale, with 0.0 indicating no evidence of infection, 1 indicating brown specks smaller than 0.5 mm in diameter, 2 indicating brown specks of 0.5–1.0 mm in diameter, 3 indicating roundish to elliptical lesions of about 1–3 mm in diameter, 4 indicating typical spindle-shaped blast lesions of 3 mm or longer with little or no coalescence lesions, whereas 5 indicating same as scale 4. However, half or more leaves were affected by coalescing blast lesions. Therefore, plants with a score of 0–3 are categorized as resistant and score of 4–5 as susceptible [37].

### 2.10. Defense Gene Expression Studies of Rice Plant upon Bacterization

After confirmation of the rice blast suppressive ability of the *M. testaceum* B2 strain, qRT-PCR studies were carried out to determine the impact of seed bacterization and booster dose sprays on the induction of defense-related genes in rice plants. Briefly, germinated PB1 and BPT5204 seeds were separately bacterized with 10^8^ and 10^7^ CFU mL^−1^ bacterial suspensions, and sample collection was conducted after seven days of bacterization. The seedlings were also planted in small pots filled with sterile soil. A booster dose of the respective bacterial suspension was delivered at the 3-leaf stage, and sampling was conducted after 24 h (before challenge inoculation by a pathogen).

Samples were quickly snap-frozen in liquid nitrogen to prevent all of the cellular metabolic activity and then stored at −80 °C until needed. The SV Tool RNA isolation system was used to isolate total RNA according to the manufacturer’s instructions (Promega Corporation, Madison, WI, USA). Spectrophotometry (NanoDrop 2000, Thermo Scientific, Waltham, MA, USA) and agarose gel electrophoresis were used to determine the quality and quantity of RNA. Rice genes associated with defense-related pathways such as *OsCEBiP*, *OsCERK1*, *OsPAD4*, *OsEDS1*, *OsNPR1*, and *OsFMO1* [38,39,40,41,42,43,44] playing a key role in rice innate immunity were selected; PCR primers targeting the above defense genes are furnished in Tables S4 and S5. qRT-PCR was performed in Real-Time Thermal Cycler (Light Cycler 96, Roche Life Science, Rotkreuz, Switzerland) using GoTaq^®^ 1-Step RT-qPCR System (Promega Corporation, Madison, WI, USA). The reaction condition for qRT-PCR is described in Table S6. Later, cyclic threshold data points were analyzed for determining gene expression relative to reference housekeeping *OsActin* gene using the software LightCycler^®^96 Roche. The mean Ct values were considered for the calculation of 2-ΔΔCT to estimate the fold changes in gene expression.

All experimental data with proper biological and technical replications were pooled and analyzed using the statistical program GraphPad Prism 9 (https://www.graphpad.com/ accessed on 31 October 2022) for one-way ANOVA and two-way ANOVA with Turkey HSD Post hoc analysis at *p* ≤ 0.05 (*), *p* ≤ 0.01 (**), and *p*≤ 0.001 (***) levels of significance. Additionally, the entire qRT-PCR data were processed using WASP-Web Agri Stat Package 2.0 to group the significant results into different groups.

### 2.11. Transcriptional Analysis of Genes Associated with Immune Competence in Rice Seedlings

#### 2.11.1. RNA Extraction, Library Preparation, and Sequencing

Having confirmed the disease suppression and defense gene activation in the rice, we tried to explore the overall transcriptional changes in rice upon bacterization. For this study, overnight soaked rice seeds (PB1) were bacterized with 10^8^ CFU mL^−1^ bacterial suspension and incubated in greenhouse conditions (Temperature 28 °C; Relative Humidity >90%; and Photoperiod 12 h). The seedlings were further transplanted in small pots filled with sterile soil after seven days and allowed to grow for three weeks. A booster dose of the same concentration of bacterial suspension was delivered at the 3-leaf stage, and the samples treated with sterile distilled water were considered as control. Then, sampling was conducted at 24 h post bacterial spray. Samples were quickly snap-frozen in liquid nitrogen to prevent all cellular metabolic activity and then stored at −80 °C until needed. The SV Tool RNA isolation system was used to isolate total RNA according to the manufacturer’s instructions (Promega Corporation, Madison, WI, USA). The quality and quantity of the RNA were accessed through the NanoDrop 2000 (Thermo Scientific, Waltham, MA, USA) and agarose gel electrophoresis. For the following library preparation, 1 μg of total RNA with a RIN value (>7) was used.

NGS libraries were prepared according to the manufacturer’s procedure (NEBNext^®^Ultra TM RNA Library Prep Kit for Illumina R). Illumina HiSeq2500 sequencing platform was used for sequencing the libraries, and the raw reads obtained after this sequencing were submitted to NCBI Sequence Read Archive under the bio-project accession number: PRJNA782087 (https://www.ncbi.nlm.nih.gov/sra/docs/submit/ accessed on 31 October 2022).

#### 2.11.2. Quality Control and Read Mapping to the Reference Genome

Public Galaxy cloud servers provide integrated tools for computation analysis workflow of NGS analysis and quality assessment; we have used them for our transcriptome analysis and workflow [45]. First, FastQC (Galaxy version 0.73) software checked the raw read quality. Then, base quality score distribution, average base content per read, and GC distribution were analyzed for the raw reads. Then, in a pre-processing step, the adaptor sequences and low-quality bases are trimmed using the Trimmomatic program (Galaxy version 0.38.1) [46]. Finally, the *Oryza sativa* (Japonica) Nipponbare genome was downloaded from MSU Rice Genome Annotation Project Database for reference-based assembly.

RNA STAR (version 2.7.8a) program was used to align the raw reads to the rice genome [47]. Cufflinks (version 2.2.1.3) were used for transcript assembly and FPKM (RPKM) estimates for RNA-Seq data [48]. Bias detection and correction can significantly improve the accuracy of transcript abundance estimates by enabling the parameters to perform bias correction–yes, library prep used for input reads fr-first-strand, exclude the records from the BAM output–unmapped bam and rest of the default parameter. Cuffcompare (version 2.2.1.2) was used to compare assembled transcripts to a reference annotation and track Cufflinks transcripts across multiple experiments.

#### 2.11.3. Differential Expression and Functional Annotation

For the differential expression analysis, Cuffdiff (v. 2.2.1.6), along with library normalization (Classic-FPKM), dispersion estimation (Pooled), and bias correction (Yes) [48]. The genes >2 log2 fold change and *p*-value < 0.05 were considered for significant change and used for further analysis. Functional annotation was retrieved from Rice Genome Annotation Project Database (http://rice.uga.edu/analyses_search_putative.shtml accessed on 31 October 2022) for the differentially expressed transcripts. VolcaNoseR was used for designing the volcano plots [49]. Selected up and downregulated genes were labeled in plots.

Versatile matrix visualization and analysis software Morpheus used for heat map (https://software.broadinstitute.org/morpheus accessed on 31 October 2022). The individual and combined unigenes samples were functionally annotated using Rice Genome Annotation Project Database. Gene ontology enrichment was performed using Database for Annotation, Visualization, and Integrated Discovery (DAVID) (https://david.ncifcrf.gov/ accessed on 31 October 2022). Further, the predicted genes were mapped for their involvement in biochemical pathways by subjecting those to pathway analysis using the Kyoto Encyclopedia of Genes and Genomes (KEGG) [50] database. Finally, the co-expression of the genes was analyzed using the String network (https://string-db.org accessed on 31 October 2022).

#### 2.11.4. qRT-PCR-Based Validation of DEGs

The DEGs shortlisted from the transcriptome data were further validated using qRT-PCR. Random selection of a few upregulated and downregulated genes was performed. Primer designing was conducted using Primer3Plus (https://www.bioinformatics.nl/cgi-bin/primer3plus/primer3plus.cgi accessed on 31 October 2022) and PrimerQuest™ Tool of Integrated DNA Technologies (IDT, Coralville, Iowa, USA) (https://www.idtdna.com/pages/tools/primerquest accessed on 31 October 2022) (Table S7). The transcriptome validation was conducted using GoTaq™ 1-Step RT-qPCR System according to the manufacturer’s (Promega Corporation, Madison, WI, USA) protocol. The rice housekeeping gene *OsActin* was used as the reference gene [20]. Data were analyzed using relative fold change calculations of the selected DEGs reference to the rice actin gene with the software LightCycler^®^96 Roche System (Roche Diagnostics International AG, Rotkreuz, Switzerland). The relative fold change in gene expressions was calculated using the 2^−ΔΔCt^ method [51].

## 3. Results

### 3.1. Polyphasic Characterization of Endophytic Bacteria

Bacterial isolate, OsEnb_ALM_B2, was isolated from rice leaves during an attempt to discover potential endophytes with probiotic properties and disease suppression properties. 16S rRNA gene sequence-based BLAST analysis revealed the isolate closely related to *Microbacterium testaceum* in NCBI GenBank. The bacterium appeared yellow, non-fluid small and round colonies with a convex surface on the NA medium. The same bacterium looked bright red with similar morphological features in redox dye-supplemented NA media (Figure S1A). TEM imaging of bacterial cells indicated typical *Microbacterium*-specific oval, rod, and V-shaped cells (Figure S1B). Biolog-based phenotypic fingerprinting confirmed its identity as *Microbacterium testaceum* (Figure S1C).

**Figure 1:**
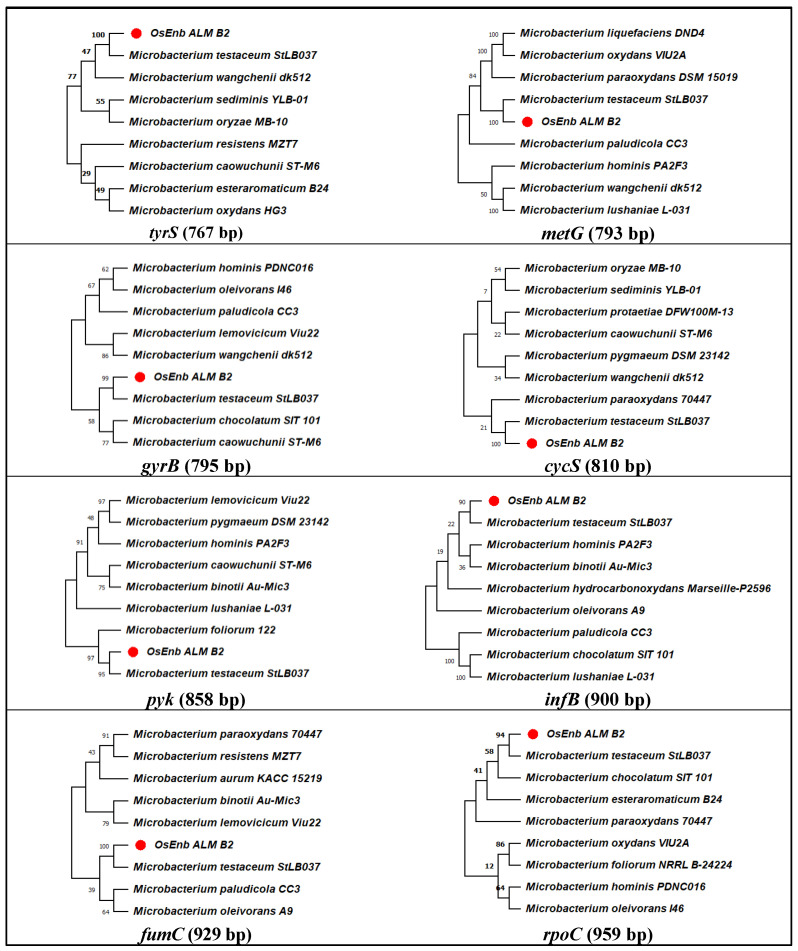
Molecular phylogenetic analysis of *Microbacterium* species by maximum likelihood method. The evolutionary history was inferred using the Maximum Likelihood method and the Hasegawa–Kishino–Yano model. The % of trees in which the associated taxa clustered together is shown above the branches. This analysis involved the nucleotide sequence of nine isolates. Evolutionary analyses were conducted in MEGA11.

Further, to reconfirm the identity of the bacterial isolate, phylogenetic analysis was conducted with eight *Microbacterium*-specific genes (Figure S1D). Curated sequences were firstly analyzed through nBLAST, which identified the isolate as *M. testaceum,* and the same was submitted to NCBI GenBank to assign accession numbers (OP419665 to OP419672). Then, species identity was further reconfirmed via phylogenetic analysis for eight gene sequences with the closely related species sequences retrieved from the database, which established the identity of endophytic isolate OsEnb_ALM_B2 as *M. testaceum* (Figure 1; Table 1 and Figure S8).

### 3.2. Evaluation of Antifungal Activity In Vitro

**Figure 2:**
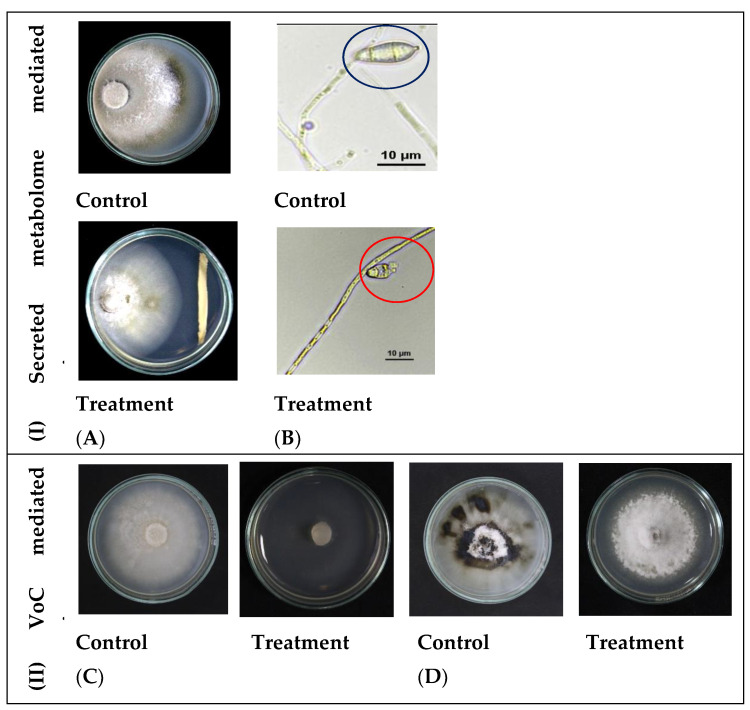
Antagonism of *M. testaceum* B2 on *M. oryzae* 1637; (I) Secreted metabolite mediated antagonism; (II) Volatile Compound mediated antagonism. Percent reduction in mycelial growth of *Magnaporthe oryzae* was observed over control in both secreted metabolite as well as volatile mediated dual culture assays. In addition, conidial morphology deformation was observed in the secreted metabolite-mediated assay (**A**) Secreted metabolites mediated inhibition of *M. oryzae*-1637. (**B**) Conidial deformation due to bacterial secreted metabolites interaction with fungus (blue circle— healthy conidia; red circle—deformed conidia). (**C**) Volatiles of *M. testaceum* B2 suppressed mycelial growth. (**D**) Volatiles of *M. testaceum* B2 showed fungistasis on mycelial growth.

The isolate *M. testaceum* B2 inhibited the rice blast fungus mycelial growth by secretory metabolome. As shown in Figure 2 and Table 2, the metabolites displayed a 19.7% mycelial inhibition compared to the control. Further, the microscopic examination of conidia revealed deformities on conidial morphology and development upon bacterial interaction. Apart from non-volatile metabolites, the volatile compounds emitted by *M. testaceum* B2 completely inhibited mycelial growth when assessed in the dual culture assay. Post-volatile exposure, the fungal mycelia reemerged, indicating that volatile-mediated inhibition is fungistatic.

### 3.3. Profiling of Volatile Organic Compounds through GC-MS

Complete mycelial growth inhibition by volatiles of *M. testaceum* prompted us for chemical profiling through GC-MS. As a result, ten volatile organic chemicals were identified in the hexane extract fraction of volatiles released by *M. testaceum* B2. Among all, three compounds, namely Acetic acid ethyl ester (31.5%), Propanoic acid ethyl ester (32.2%), and Hexadecanoic acid (12.3%), were found to be predominant in the profile. On the other hand, the proportion of other minor compounds ranged from 0.28 to 6.3% (Figure S2; Table 3).

### 3.4. Evaluation of Potential Pathogenic Nature

Tobacco hypersensitivity assay suggested that the bacterium *M. testaceum* B2 is not pathogenic since no necrosis was seen even after 48 h of infiltration, while *Ralstonia solanacearum* caused prominent wilt lesions upon infiltration (Figure S3).

### 3.5. Studies on Endophytic Colonization of Microbacterium testaceum B2 in Rice Plant

#### 3.5.1. GFP Transformation of Bacterium

**Figure 3:**
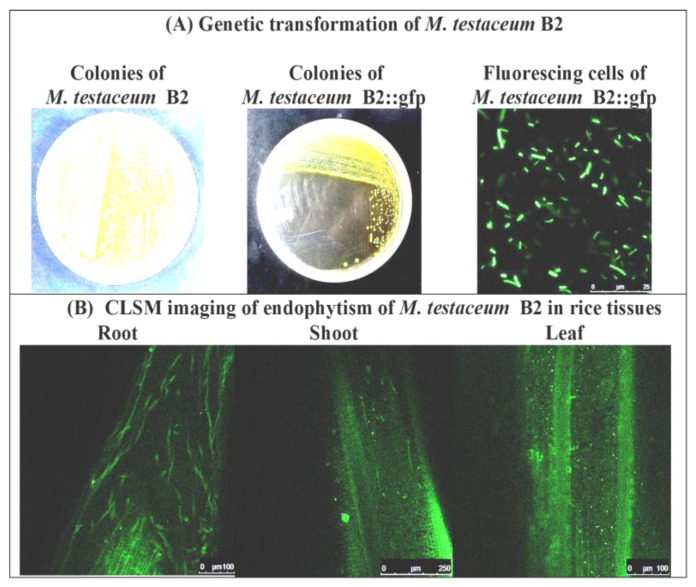
Endophytism of *Microbacterium testaceum* B2::gfp in rice plants as revealed by confocal laser scanning microscopy.(**A**) Genetic transformation of *M. testaceum* B2 using Tn7-based neutral integration of gfp gene. (Images from left to right: Intrinsic rifamycin resistant colonies on NA+Rif50 plate, gfp transformed colonies on NA+Rif50+Gm20 plate, pure culture cells of transformed *M. testaceum* B2::gfp).(**B**) CLSM imaging of endophytically colonized *M. testaceum* B2::gfp.

Intrinsic rifamycin-resistant colonies (Figure S4) of bacteria were used for GFP transformation using the triparental mating method, and the double antibiotic-resistant plate (rifamycin + gentamycin) was used for the selection of putatively transformed cells. Multiple transformed colonies appeared on the selection plates, further confirmed with PCR and confocal laser scanning microscopy (CLSM) for gene integration and stable expression (Figure 3A and Figure S5). Colonies with positive PCR reactions and excellent fluorescent expression under CLSM were selected and preserved for long-term use as glycerol stock. The GFP derivative of isolates was thus designated as *M. testaceum* B2R::gfp having features such as dual antibiotic resistance, and excellent GFP expression was used to study endophytic colonization in rice plants.

#### 3.5.2. Endophytic Colonization on Rice

Bacterial isolate *M. testaceum* B2R::gfp colonized the rice tissue (both above and below) upon treatment at the germination stage, as confirmed by confocal microscopy and PCR-based detection. Surface sterilized seedlings were first fixed with *p*-formaldehyde (4%), and then thin sections of tissue were made, followed by their visualization under CLSM. Stable gfp expression by bacterial cells inside the plant tissue reflected its endophytic colonization (Figure 3B). Endophytic colonization was also confirmed by PCR using GFP-specific primers, where an amplicon of 142 bp was obtained from treated plants, while no amplification was recorded in untreated control plants (Figure S6).

### 3.6. Plant Probiotic Features of Microbacterium testaceum B2

**Figure 4:**
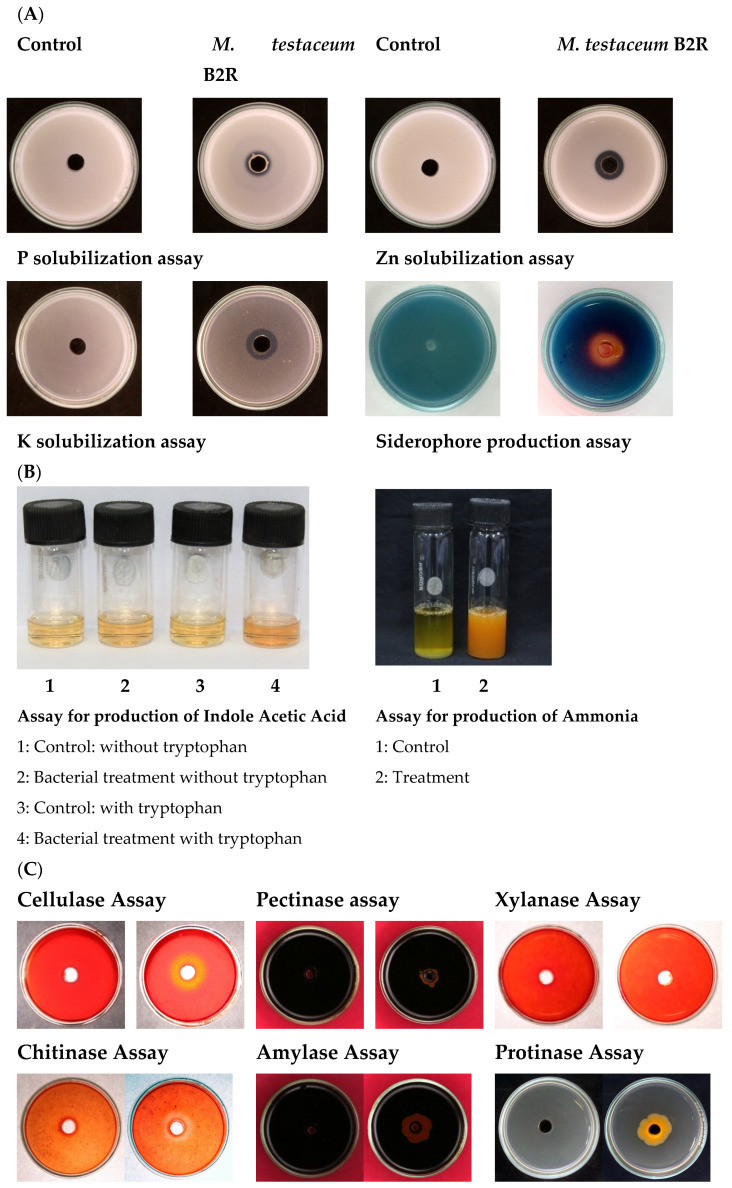
Plant probiotic features of *M. testaceum* B2 (**A**) Mineral solubilization assays. (**B**) Production of IAA and Ammonia. (**C**) Production of hydrolytic enzymes.

*M. testaceum* B2 showed multiple plant probiotic features, such as the production of enzymes, phytohormones, and mineral solubilization. The bacterium showed Zn, P, and K solubilization activity and produced auxin (IAA), ammonia, and siderophore under in vitro conditions. Further, we observed some enzymatic activity such as cellulose and chitinase production by the rice endophyte, like many biological control agents. Moreover, it showed a negative result for other enzymes such as xylanase, amylase, pectinase, and proteinase (Figure 4; Table S9).

### 3.7. Microbacterium testaceum B2-Mediated Priming in Seedlings

**Figure 5:**
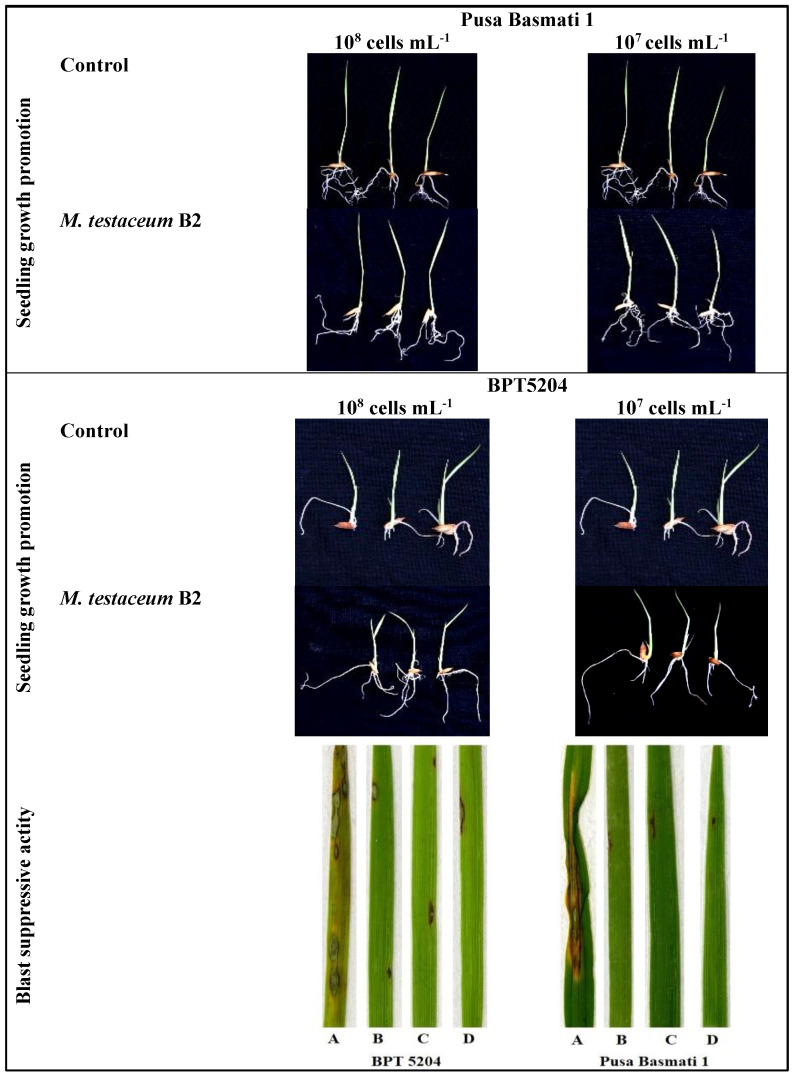
*M. testaceum* B2 induced growth promotion in rice seedlings and blast disease suppressive activity. *M. testaceum* stimulated the growth of rice seedlings upon bacterization; the number of lesions and size of lesions were found to be reduced in bacterized plantlets; plant responses are scored as per Mackill and Bonnman [37]. (**A**) Control (**B**) *M. testaceum* B2 (10^8^ CFU mL^−1^). (**C**) *M. testaceum* B2 (10^7^ CFU mL^−1^). (**D**) Tricyclazole.

The bacterial primed seeds showed complete (100%) germination. Among the seedling characteristics, maximum shoot length was observed on PB1 seedlings treated with bacterial suspension (10^7^ cells mL^−1^). The same treatment also exerted a maximum number of roots and fresh weight in rice seedlings. On the other hand, the highest number of roots was observed in BPT5204-treated with 10^7^ cells mL^−1^ of *M. testaceum* B2. All the treatments showed a significant positive effect on rice plants’ seed germination and initial seedling growth, irrespective of the cultivar (Figure 5; Table 4).

### 3.8. Evaluation of Disease Control Efficacy

Two blast-susceptible rice cultivars (Pusa Basmati 1 and BPT5204) were used to testify the *M. testaceum* B2 disease suppression activity in the form of direct seedling treatment and foliar spray at 10^7^ and 10^8^ CFU mL^−1^. The biocontrol bacterial agent showed good suppression (more than 78% in all treatments) of blast disease under greenhouse conditions at both tested doses. The disease severity was significantly less than the control plants in all treatments. Maximum disease suppression was observed at a lower dose, i.e., 10^7^ CFU mL^−1^ in both the cultivars (90.6% in PB1 and 85.5% in BPT-5204) (Figure 5; Table 4).

### 3.9. Evaluation of Immunocompetence Conferred by Microbacterium testaceum B2

**Figure 6:**
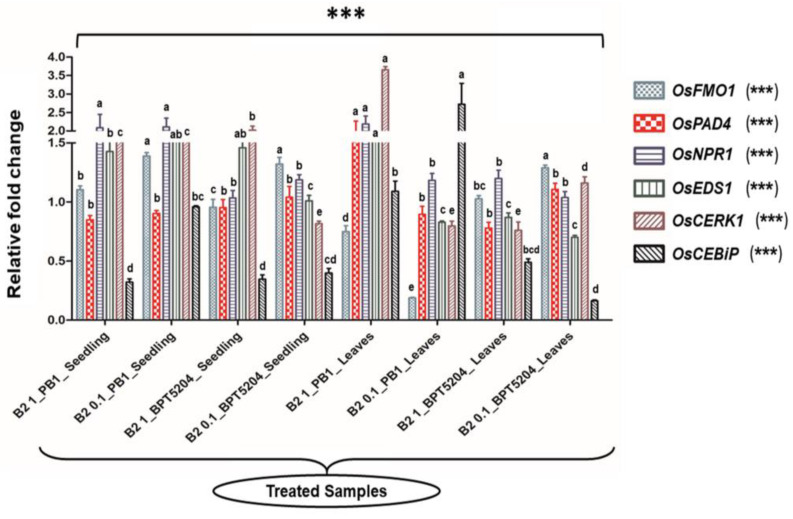
Transcriptional response of defense genes in rice upon bacterization by *M. testaceum* B2. qPCR-based transcriptional analysis of defense gene expression in rice seedlings upon endobacterization. The fold change values obtained for the defense genes were imported into the GraphPad Prism program (https://www.graphpad.com/scientific-software/prism accessed on 31 October 2022), and two-way ANOVA was conducted using Bonferroni posthoc test for determining the statistical significance at *** *p* = 0.0001. Refer to Table S10 for data on fold changes in gene expression. (The data designated with different alphabets were significantly different. The letter stands for non-significant.).

Microbes can activate the defense-related pathways in the host plant. Having confirmed the disease suppression activity, a detailed investigation was made to evaluate the gene expression pattern upon bacterial treatment. The bacterized rice seedlings and booster dose-treated rice leaves were analyzed for several defense-related genes (*OsCERK1*, *OsCEBiP*, *OsNPR1, OsPAD4*, *OsEDS1*, and *OsFMO1*) through the qRT-PCR technique. They showed low to marginal upregulation on the bacterized seedlings compared to the untreated control. Both rice cultivars showed a varied pattern of gene expression after the bacterial treatment. In the PB1 cultivar, the *OsEDS1* gene was significantly upregulated in almost all the tested doses, while the *OsCERK* gene was highly upregulated in leaves tested with 10^8^ CFU mL^−1^ bacterial dose. The *OsCEBiP* gene was upregulated in the leaves upon treatment with a titer of 10^7^ CFU mL^−1^. In the case of the BPT5204 cultivar, the*OsCERK1* gene was upregulated in the bacterized seedling tested with 10^8^ CFU mL^−1^ (Figure 6; Table S10).

### 3.10. RNA-Seq Data Statistics

The Illumina HiSeq 2000 platform-based RNA-sequencing yielded an average of 3.3–5.6 million reads with an average read length of 150 bp. When the sequences were aligned with the rice reference genome, the mapping % varied from 87 to 88%. The transcriptome assembly statistics after processing have been tabulated in Table S11. The reliability and quality of RNA seq data were assured after considering the Phred score value ≥ Q35. A total of 24,028 and 24,187 genes were found in Control and treated samples, respectively. Around 85% of genes were common in both samples, and 7% were exclusively found in both control and treatments (Figure S7). The RNA-seq data were deposited at the NCBI Sequence Read Archive (SRA) database under bio-project number (PRJNA782087).

### 3.11. Functional Analysis of Induced DEGs

A total number of 19,128 genes were found through the Cuffdiff program. Further up- and downregulated genes were shortlisted based on a *p*-value of 0.05 and ± 2 log2 fold change, which was considered for significant change. We got 62 upregulated and 18 downregulated genes (Tables S12 and S13). After functional annotation analysis using Rice Genome Annotation Project, 20 genes were shortlisted based on defense response, plant hormone, and secondary metabolite pathways for the qRT-PCR primer designing and transcriptome data validation (Figure S8; Table S7). The differential gene expression volcano plot was mapped using log2 fold change expression parameters and –log10 *p*-value. The *p*-value cut-off value < 0.05 was considered for the significant change (Figure S9).

### 3.12. GO Classification

The DEGs were further classified for the GO terms. GO-enrichment of upregulated genes suggested that most of the genes were involved in the metabolic process (GO: 0008152), cellular process (GO: 0009987), response to stress (GO: 0006950), response to abiotic stimulus (GO: 0009628), and secondary metabolic process (GO: 0019748) under the GO type biological process. Under cellular components genes are involved in the membrane (GO: 0016020), cell (GO: 0005623), plasma membrane (GO: 0005886), cytosol (GO: 0005829) and cell wall (GO: 0005618); catalytic activity (GO: 0003824), transferase activity (GO: 0016740), hydrolase activity (GO: 0016787), nucleotide binding (GO: 0000166), and binding (GO: 0005488) under the GO type molecular function.

**Figure 7:**
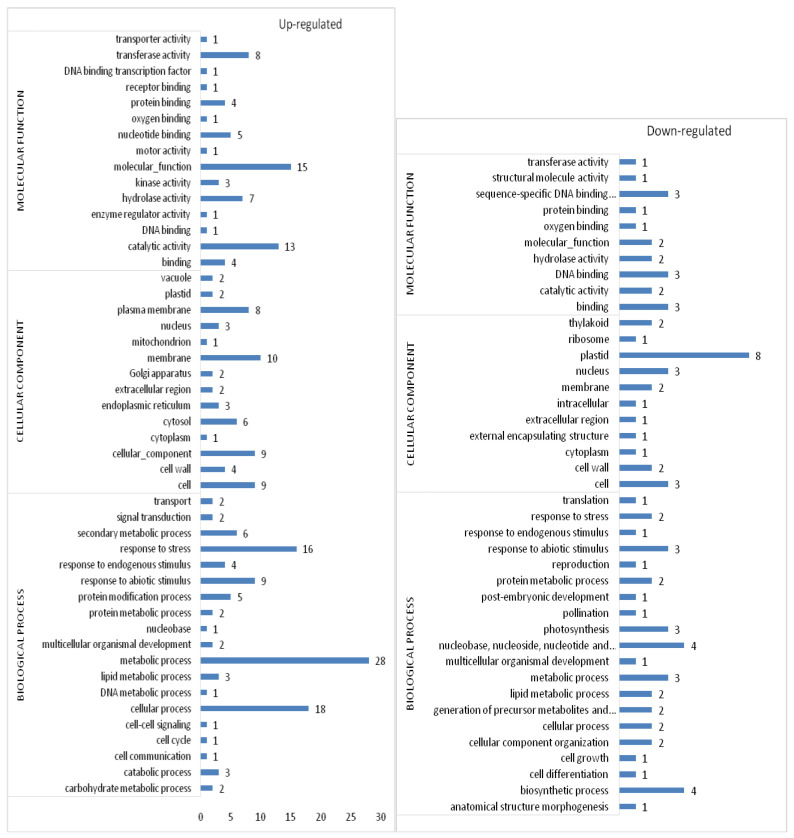
GO classification of differentially expressed up- and downregulated genes. The genes involved in biological process, cellular component, and molecular function. The x-axis indicates the number of genes involved.

**Figure 8:**
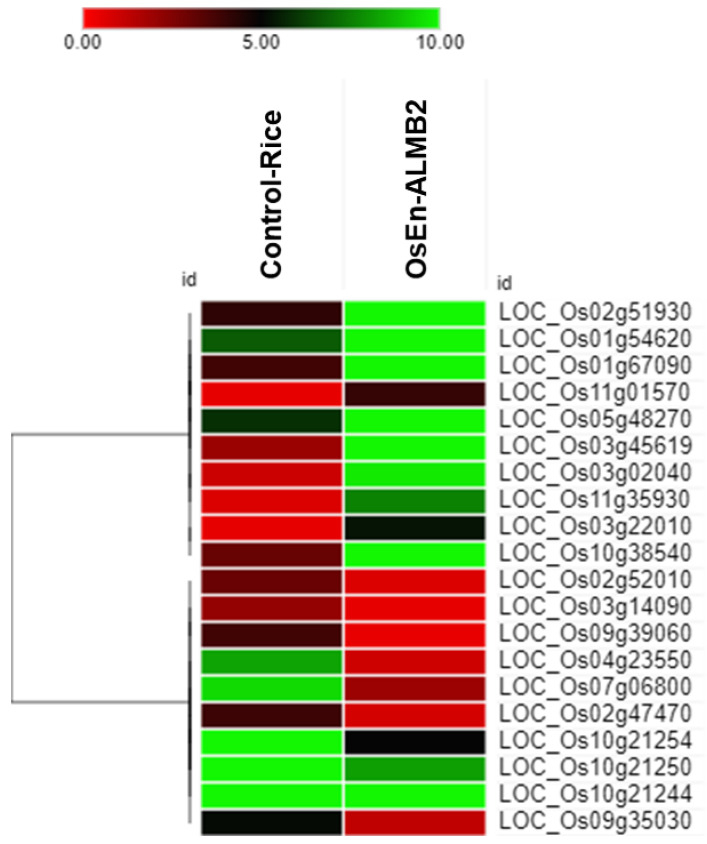
Heat map plotted with absolute FPKM expression value.

Similarly, GO enrichment of downregulated genes is suggestive of several pathways such as biosynthetic process (GO: 0009058), nucleobase, nucleoside, nucleotide and nucleic acid metabolic process (GO: 0006139), metabolic process (GO: 0008152), photosynthesis (GO: 0015979) and generation of precursor metabolites and energy (GO: 0006091) under the GO type biological process; plastid (GO:0009536), cell (GO:0005623), nucleus (GO: 0005634), membrane (GO: 0016020) and thylakoid (GO: 0009579) under the GO type cellular component; DNA binding (GO:0003677), sequence-specific DNA binding transcription factor activity (GO: 0003700), catalytic activity (GO: 0003824), hydrolase activity (GO: 0016787), and protein binding (GO:0005515) under the GO type molecular function (Figure 7). Furthermore, the heat map revealed the upregulation and downregulation of significant DEGs, which are essential in the abovementioned pathways (Figure 8).

The co-expression study of DEGs via String network analysis showed their interactions with other proteins. Os01g54620 (CESA4) is Cellulose synthase A catalytic subunit 4 co-expressed with other proteins involved in COBRA-like protein 5, peroxidase removal H2O2. Os02g47470 (CYP707A5) is Abscisic acid 8′-hydroxylase 1, which is involved in the oxidative degradation of abscisic acid. Os03g02040 (Q10SU0) is a Remorin C-terminal region family protein that interacts and co-expressed with Latex-abundant protein, Nitrate-induced NOI protein, Tubulin beta-8 chain, and Importin subunit alpha. Os03g22010 (prx41) peroxidase, Removal of H2O2, oxidation of toxic reductants, biosynthesis and degradation of lignin, suberization, auxin catabolism interact with chlorophyllide an oxygenase, chlorophyll synthase, and protein thylakoid formation. Os03g45619 (Q10FR4) cytochrome P450 family Pyrophosphate--fructose 6-phosphate 1-phosphotransferase subunit alpha co-expressed with Ent-cassadiene C11-alpha-hydroxylase 1, Protease inhibitor, Pyrophosphate-fructose 6-phosphate 1-phosphotransferase subunit alpha, and Transposon protein. Os09g35030 (DREB1A) dehydration-responsive element-binding protein 1A interacts with Homeobox-leucine zipper protein HOX17, Molybdenum cofactor sulfurase, Molybdenum cofactor sulfurase, belongs to the disease resistance NB-LRR family and Repressor of jasmonate responses. Os10g38540 (Q945W4) Glutathione S-transferase GSTU6 also participates with Ent-sandaracopimaradiene 3-hydroxylase, Lignin degradation and detoxification of lignin-derived products, Xylanase inhibitor protein 1 and Helix-loop-helix DNA-binding domain containing protein. Os11g01570 (XOAT12) Xylan O-acetyltransferase co-expressed with Ferritin and Ferredoxin-thioredoxin reductase catalytic chain. Os11g35930 (Q2R2G0) Chalcone and stilbene synthases co-expressed with Probable 4-coumarate--CoA ligase 1, Probable 4-coumarate--CoA ligase 4, Hydroxycinnamoyltransferase 1, and Glycosyltransferase (Figure S10).

## 4. Discussion

As an evolutionary rule, higher eukaryotes such as plants and animals have not evolved independently but coevolved with many endophytic and epiphytic microbiota. Among them, endophytes are more significant since they colonize the plant’s internal tissue without causing harm to the host. The plant itself recruits the majority of the endophytes in response to a specific environmental factor, such as biotic or abiotic stress [52].

In the current work, we explored the beneficial effect of rice endophytic bacterium (*Microbacterium testaceum* isolate OsEnb-ALM-B2) for possible biological control of rice blast disease. To establish the bacterial identity, we adopted polyphasic taxonomic tools. On the nutrient medium, the bacterium looked pale yellow-colored, small, non-mucoid, round, and convex, whereas they appeared dark red in redox dye-amended nutrient agar plates. Transmission electron microscopic imaging of cells reflected cell structures that were short slender rods occurring singly or in groups. Bacterial identity at the species level was confirmed through 16S rRNA gene sequence analysis and multi-locus sequence analysis (*tyrS, rpoC, fumC, pyk, metG, infB, gyrB,* and *cysS*) [53]. *Microbacterium testaceum* was the closest match in all cases according to BLAST analysis on the NCBI database. Further above, genes used for phylogenetic analysis along with the reference sequences retrieved from the database also revealed their identity as *M. testaceum* as the rice endophyte clustered with potato leaf endophyte *M. testaceum* StLB037 [54]. In the past, *M. testaceum* has been observed in several ecological niches such as river water [55], forest soil [56], animal feed [57], industrial wastewater [58], and as well as an endophyte in corn, sorghum, potato, rice, etc. [23,54,59]. In addition, biosafety concern is paramount when introducing novel microbial inoculants into agronomic practice [60,61]. *M. testaceum*OsEnb-ALM-B2 appeared non-pathogenic as no HR reaction occurred on tobacco leaves. Gfp tagging of microbes has become one of the efficient genetic tools for visualizing endophytic colonization in plant tissue [19]. Therefore, we genetically transformed the bacterium with the GFP gene and studied endophytic colonization and population estimation. As a result, the bacterium could be visualized in both above- and below-ground plant tissues.

In order to qualify as a successful bioinoculant in agriculture, the microbial species must display plant growth promotion and pathogen-suppressive activities. *M. testaceum* B2 showed positive for Zn, P, and K solubilization and produced ammonia, siderophore, and auxin (IAA). Zn is an essential micronutrient for plants and humans as it is required to complete several metabolic processes. Zn deficiency may cause a severe reduction in growth and is mainly involved in the synthesis of IAA, thus hampers crop yield [62]. Further, nutrition devoid of Zn may lead to retarded growth and impairment of physical health in human beings [63]. Thus, an increased supply of Zn to plants via microbial inoculant may serve dual purpose benefits for the crop and human health.

P and K are among the essential nutrients a plant needs for its growth and are supplied in bulk through NPK fertilizers [64]. However, most Indian soils are deficient in K [65]. Therefore, *M. testaceum* B2-aided solubilization of K is expected to enhance plant growth and development and partially reduce the dependency on chemical fertilizers. On the other hand, a large portion of the soil P is in an “insoluble state” and unavailable to plants. Despite the widespread use of chemical fertilizers, P levels in soil worldwide are currently declining; hence, microbe-mediated P solubilization may increase its plant availability. In the recent past, microbe-mediated P solubilization has been enumerated by various researchers [66,67].

Similarly, ammonia production by endophytes contributes to the N nutrition of the plants [68]. Endophyte-mediated N supplement is considered better than their rhizospheric counterparts as N is directly available to the plant and bypasses competition with other soil organisms [69]. In addition, Fe is an essential mineral nutrient that is needed by most plants and their pathogens [70]. Fe chelation by an endophyte through the production of a siderophore is a dual-purpose mechanism where Fe becomes unavailable for the pathogenic organism and more available to plants for its growth and development [71]. In our case, *M. testaceum* B2 also appeared prolific for ammonia and siderophore production. Aznar and Dellagi [72] have recently indicated that JA and SA signaling-mediated defense mechanisms can also be triggered through siderophore, which might contribute to plant immunity against biotic stresses. Just like minerals, isolate B2 also displayed the IAA production. Thus, produced IAA might be contributing to enhancing cell proliferation, elongation, and root development [73]. The results suggest that *M. testaceum* B2 promotes the biological availability of significant nutrients and hormones and indirectly enhances rice plant growth. *M. testaceum* B2 also tested positive for producing hydrolytic enzymes such as cellulase and chitinase. Chitinolytic activity is particularly relevant for a biocontrol agent with antifungal traits against chitin-containing plant pathogenic fungi [74]. On the other hand, cellulases are essential for bacterial colonization and spread inside the plant tissue [75]. We observed a positive effect on early seedling growth in terms of increased germination %, increased biomass, and increased shoot and root length, which has been noted earlier with other microorganisms involved in plant growth promotion [68,76].

Most importantly, excellent in vitro antifungal activity has been shown by the metabolites of rice endophyte *M. testaceum* B2 during interaction with the blast fungus. Non-volatile compound-mediated interaction also resulted in the deformation of the conidial structure apart from mycelial growth inhibition. Mycelial growth was completely inhibited by volatile compounds, which were further characterized as fungi-static. Recently, bacterial volatile and non-volatile compound-mediated suppression of rice blast fungus has been demonstrated by Sahu et al. [12]. Before the present study, volatile compounds secreted by *Pseudomonas putida* BP25 had also been reported to inhibit multiple phytopathogenic fungi under in vitro conditions [19].

Seeing the potential of bacterial native volatiles, we further performed chemical characterization using a GC-MS system that revealed a high abundance of three organic compounds (acetic acid ethyl ester, propanoic acid ethyl ester, and hexadecanoic acid). The antimicrobial activity of acetic acid ethyl ester has been reported by Ates et al. [77]. Similarly, propanoic acid ethyl esters were synthesized and proved to have an inhibitory effect against fungal and bacterial pathogens [78]. Hexadecanoic acid is a fatty acid naturally present in several plants with antibacterial, antifungal, and anti-inflammatory properties [79,80,81,82]. Additionally, the antifungal nature of many other volatile chemicals released by *M. testaceum* B2 has already been reported in the literature, for example, 2-Hexanone [83,84], 3-Hexanol [85], 9-Octadecenoic acid [86,87], and Eicosanoic acid [88].

Both PB1 and BPT 5204 have been used for a long time as susceptible checks during rice blast resistance screening programs [89,90]. We used these lines to assess the blast suppressive activity of *M. testaceum* B2 under greenhouse conditions. The seedlings primed with endophytic bacteria significantly reduced disease severity compared to the untreated plants. Before the present report, *Microbacterium oleivorans*-mediated suppression of rice blast disease under greenhouse conditions has been demonstrated by Sahu and colleagues [12]. In another report, Wang et al. [91] also reported disease suppression activity by potato endophyte *M. testaceum* StLB037 against potato soft rot pathogen. Based on the literature survey, the biocontrol activity of endophytic *M. testaceum* B2 against rice blast disease is novel. The blast disease suppression could be attributed to the *M. testaceum* elicited defense pathway in rice plants, as noted in the qRT-PCR-based gene expression analysis that revealed upregulation for most of the studied genes. Genes significantly expressed due to bacterial treatment were *OsCEBiP*, *OsCERK1,* and *OsEDS1*. Whereas *OsCERK1* is a receptor-like kinase responsible for the perception of MAMP molecule chitin and peptidoglycan along with a receptor-like protein *OsCEBiP* [38,39], the*OsEDS1* is a marker for induced systemic resistance (ISR) mediated by phytohormone Jasmonic acid [41]. The disease-suppressive potential of a microbial agent is attributed to multipronged antifungal antagonism, niche saturation, and elicitation of host defense [21]. After generating preliminary information about the induction of selected defense-related genes, we further analyzed the complete gene-expression profile of rice plants upon bacterization via high-throughput transcriptome sequencing. In particular, we explored the transcriptome data for defense genes responsible for protection against biotic stresses in general and against fungal pathogens. The analysis of differentially expressed genes (DEGs) revealed that 80 genes were differentially expressed significantly upon rice– *M. testaceum* B2 interaction. Among them, 62 were upregulated, and 18 were found to be downregulated. These DEGs were then sub-categorized based on their function into housekeeping and defense-related functions. Most of the upregulated genes were known to have protective functions against biotic stresses. Upregulated genes such as*Os11g35930* code for chalcone synthase (CHS) directly contribute to the production of phenolic phytoalexins in rice [92]. Different phenolic phytoalexins are produced in rice upon biotic and abiotic stimuli. Moreover, Sakuranetin is a well-characterized phytoalexin that protects the plant against blast disease [93].

Gene *Os11g01570* is a Trichome Birefringence-Like (OsTBL2) protein responsible for the acetylation of xylans (hemicellulose). This modification in cell wall polymers such asxylan imparts resistance against the leaf blight disease of rice [94]. Another over-expressed gene, *Os10g38540,* codes for glutathione S-transferase (GST), which helps protect the cell from biotic and abiotic stressors, such as pathogen invasion and heavy metal invasion toxicity, and oxidative stress [95]. There are many reports that GST also has a role in stress signaling [96] and inhibiting apoptosis [97].

Peroxidases are a set of enzymes associated with forming a secondary physical barrier, such as lignin, and protecting the plant against pathogen invasion [98]. Upregulated genes such as*Os03g13200*, *Os03g22010*, and *Os05g04500* codes for peroxidases that indicated the activation of defense-related pathways by *M. testaceum* B2. Remorins (*Os08g36760* and *Os03g02040*) are cellular proteins responsible for signal transduction. They are generally activated during biotic interactions and protect the plant from bacterial infections [99]. Upregulation of cellulose synthase (*Os01g54620*) and transferase family protein (*Os06g39390*) are mainly responsible for cellulose synthesis [100] and cross-linking of cell wall material [101], respectively. These two directly indicate stress management in the plant by forming a protective barrier. It prevents the entry of pathogens inside the plant cell and plays an essential role in the host defense mechanism [102].

Overexpression of Rapid Alkalinization Factor (RALF,*Os11g26190*), a cell signaling protein, points towards rapid alkalinization of extracellular space by increasing cytoplasmic Ca^2+^ concentration followed by Ca^2+^-dependent signaling events such as activation of mitogen-activated protein kinases (MAPK) [103]. Much literature suggests that there is a tradeoff between plant growth and defense. Whenever a defense pathway is activated or elicited by internal/external factors, there is a compromise in growth and development-related pathways [104].

In the transcriptome analysis, we further observed downregulation of some genes related to typical plant developmental processes; these genes are *Os02g52010* (phosphate-induced protein 1), *Os03g14090*(armadillo/beta-catenin repeat family protein), *Os04g23550* (basic helix-loop-helix family protein), *Os10g21244* (chloroplast 30S ribosomal protein), *Os10g21248* (photosystem I P700 chlorophyll an apoprotein A2), *Os10g21250* (photosystem I P700 chlorophyll an apoprotein A1), and *Os10g21254* (photosystem I assembly protein ycf3). *Os10g21248*, *Os10g21250*, and *Os10g21254* are genes coding for proteins associated with photosystem I chlorophyll and directly involved in photosynthesis [105].

Gene *Os02g52010* (phosphate-induced protein 1) is involved in the signaling process of regulating cell division and differentiation through modulating hormonal responses [106]. *Os03g14090* (armadillo/beta-catenin repeat family protein) is involved in cell differentiation and lateral root development [107]. *Os04g23550* (basic helix-loop-helix family protein) are family of transcription factors involved in the expression of many proteins related to plant physiological processes [108]. *Os10g21244* (chloroplast 30S ribosomal protein) is involved in protein synthesis for fulfilling the internal requirement of chloroplast proteins [109].An essential fundamental of plant economics states that to survive and reproduce in nature, plant growth and defense routes must balance one another [110]. The results of our transcriptome analysis with bacterized rice seedlings support the same phenomena at the molecular level. Our polyphasic characterization of *M. testaceum* B2, including transcriptome analysis, revealed multipronged beneficial activities that ultimately led to a novel bioinoculant for organic rice farming.

## Figures and Tables

**Table 1:** Identity of endophytic *Microbacterium* based on the closest match of multiple gene sequences.

Gene Name	Seq. Length (bp)	Max Score	Total Score	Query Cover (%)	E- Value	Identity (%)	* Closest Match	Species Identity	Accession Number Assigned
B2_*tyrS*	760	1166	1166	100	0.0	94.3	AP012052.1	*Microbacterium testaceum*	OP419672
B2_*metG*	784	1238	1238	100	0.0	95.1	AP012052.1	*Microbacterium testaceum*	OP419671
B2_*gyrB*	785	941	941	100	0.0	88.3	AP012052.1	*Microbacterium testaceum*	OP419665
B2_*cycS*	800	985	985	100	0.0	88.8	AP012052.1	*Microbacterium testaceum*	OP419668
B2_*pyk*	842	1334	1334	100	0.0	95.2	AP012052.1	*Microbacterium testaceum*	OP419670
B2_*infB*	887	1506	1506	100	0.0	97.2	AP012052.1	*Microbacterium testaceum*	OP419666
B2_*fumC*	908	1317	1317	100	0.0	92.8	AP012052.1	*Microbacterium testaceum*	OP419669
B2_*rpoC*	935	1567	1567	100	0.0	96.9	AP012052.1	*Microbacterium testaceum*	OP419667

* Gene sequences were BLAST analyzed in GenBank Database https://blast.ncbi.nlm.nih.gov/Blast.cgi?PROGRAM=blastn&PAGE_TYPE=BlastSearch&LINK_LOC=blasthome; accessed on 1 January 2020.

**Table 2:** *Microbacterium*-mediated inhibition of *Magnaporthe oryzae* upon bacterium–fungus interaction.

Treatment	Mycelial Growth Inhibition (%)		
	Secreted Metabolite	Volatile Organic Compounds	
		Exposure Effect	Release Effect
Control	0	0.00	0.00
OsEnb_ALM_B2	19.79	100.00	10.83

**Table 3:** Volatile organic compounds *Microbacterium testaceum* B2 as analyzed in GC-MS.

Compound Name *	Retention Time (min)	Area (%)
**Acetic acid ethyl ester**	**3.59**	**31.59**
**Propanoic acid, ethyl ester**	**4.65**	**32.29**
11-Pentadecenal	5.15	3.49
3-Buten-2-one	5.24	2.65
2-Hexanone	6.48	4.26
2,3-Epoxyoctan-1-ol	6.99	6.38
3-Hexanol	9.10	5.84
**Hexadecanoic acid**	**21.29**	**12.39**
9-Octadecenoic acid	28.62	1.52
Eicosanoic acid	31.55	0.28

* The most abundant ones are highlighted here.

**Table 4:** Growth stimulant and Blast disease suppressive activity of *Microbacterium testaceum* B2 on rice (**A**) Pusa Basmati 1. (**B**) BPT5204.

(A)									
Bacterial Dose (CFU mL^−1^)	Germi-Nation (%)	*Shoot Length (cm)	Root Length (cm)	Number of Roots	Fresh Weight (g)	Dry Weight (g)	Net Weight (g)	Percent Disease Index	Reduction in Disease Severity (%)
Control	100	4.77c	4.23c	3.67	0.36c	0.15c	0.22c	48.03	
10^8^	100	5.07b	5.47a	3.33	0.43b	0.17a	0.26b	7.86	83.64
10^7^	100	5.60a	4.53b	4.67	0.56a	0.16b	0.40a	4.47	90.68
CD(0.05)	NS	0.15	0.12	NS	0.004	0.002	0.004		
CV		1.45	1.22	14.43	0.46	0.66	0.72		
(B)									
Bacterial Dose (CFU mL^−1^)	Germi-nation (%)	* Shoot length (cm)	Root length (cm)	Number of roots	Fresh weight (g)	Dry weight (g)	Net weight (g)	Percent Disease Index	Reduction in Disease severity (%)
Control	100	3.07b	4.13b	2.67	0.28b	0.11a	0.18b	45.71	
10^8^	100	3.03b	6.03a	3.67	0.31a	0.07b	0.19b	9.86	78.43
10^7^	100	3.60a	3.60c	3.67	0.29b	0.10a	0.21a	6.61	85.53
CD(0.05)	NS	0.15	0.22	NS	0.01	0.01	0.01		
CV		2.31	2.41	17.32	2.19	3.0	3.66		

* M. testaceum B2 induced growth alteration in rice seedlings treated with 10^8^ and 10^7^ CFU mL^−1^, bacterial suspension after seven days. (The data with the same alphabet in each column were significantly similar to each other, but the data with different alphabets were significantly different. The letter ns stands for non-significant.).

## Data Availability

The transcriptome raw reads with Bio-project Accession no. PRJNA782087 (SRA Submission ID SUB10652121) is available in NCBI GenBank. Other information is available in the present MS and Supplementary Materials.

## Supplementary Materials

The following supporting information can be downloaded at:https://www.mdpi.com/article/10.3390/microorganisms11020362/s1, Table S1. Details of primers used in the study for genetic characterization of *Microbacterium testaceum* D18. Table S2. PCR reaction mixture for amplification of *M. testaceum*-specific genes. Table S3. PCR temperature condition for amplification of *M. testaceum*-specific genes. Table S4. Rice defense genes used for the qPCR analysis and their function. Table S5. List of the PCR primers used in the gene expression studies. Table S6. Reaction condition for one-stepqRT-PCR reaction. Table S7. Primers used for validation of transcriptome data. Table S8. List of bacterial species used in molecular phylogenetic analysis. Table S9. Plant probiotic traits of *Microbacterium testaceum* B2. Table S10. Expression analysis of defense-related genes in rice upon bacterization by *Microbacterium testaceum* B2. Table S11.RNA-Seq Data Statistics. Table S12- List of upregulated DEGs. Table S13 List of downregulated DEGs. Figure S1. Characterization and identification of endophytic *Microbacterium testaceum* B2. (A)Colony colour and pigmentation of *Microbacterium testaceum* B2, (B)TEM images of *Microbacterium testaceum* B2, (C) Phenotypic fingerprinting of *Microbacterium testaceum* B2, (D) Multigene amplicon profile of *Microbacterium testaceum* B2. Figure S2. GC-MS chromatogram profile of *Microbacterium testaceum* B2. Figure S3. Assay for pathogenic ability of *M. testaceum* B2R by hypersensitivity assay on *N. tabacum* by leaf infiltration. (A) Leaf infiltrated with sterile distilled water (Negative control). (B) Leaf infiltrated with *M. testaceum* B2R. (C) Leaf Infiltrated with *Ralstonia solanacearum* (Positive control). Figure S4. Selection of rifamycin-resistant *Microbacterium testaceum* B2. Figure S5. Genetic transformation of *M. testaceum* B2 for gfp expression. Figure S6. Agarose gel image showing the PCR amplicon of gfp gene inserted in *M. testaceum*. Figure S7. Total number of genes obtained in Control-Rice and OsEn-ALM_B2-treated rice. Figure S8. Relative fold change of the up- and downregulated genes in rice upon colonization by *Microbacterium testaceum* OsEnb_ALM_B2. The columns represent the means of biological replicates ± SE. The overall significant differences between treatments are represented by *p* ≤ 0.001 (***) as per Tukey’s post hoc HSD analysis. The data designated with the same alphabet were significantly similar, whereas the data with various alphabets were significantly different from each other. Treatments were found significant at 5% level of significance and critical difference of 0.05. Figure S9. Differential genes expression volcano plot mapped with Log2 fold change expression and –Log10 *p* value. The *p* value cut-offs were <0.05 and were considered for the significant change. Figure S10. Coexpression analysis of DEGs through String network. References [16,20,27,38,39,40,41,42,43,44,111] are cited in the supplementary materials.
