# Supplementary material for: New Insights on Endophytic Microbacterium-Assisted Blast Disease Suppression and Growth Promotion in Rice: Revelation by Polyphasic Functional Characterization and Transcriptomics
Source: Microorganisms. 2023 Jan 31;11(2):362. doi: 10.3390/microorganisms11020362 (PMC9963279; doi:10.3390/microorganisms11020362)
Supplement: Supplementary file 1 [file microorganisms-11-00362-s001.zip › microorganisms-2043028-Supplementary Figures.pdf]

Colonies of Rice Endophytes		Close up view of colonies		
Nutrient Agar	Nutrient agar + 2, 3, 5 triphenyl tetrazolium chloride	Nutrient Agar	Nutrient agar + 2, 3, 5 triphenyl tetrazolium chloride	**(A) Colony colour and pigmentation of *Microbacterium testaceum* B2**
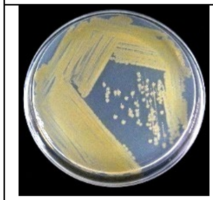	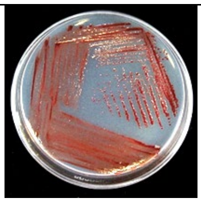	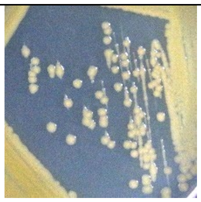	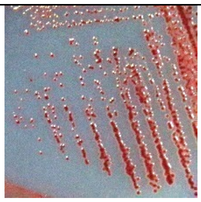	

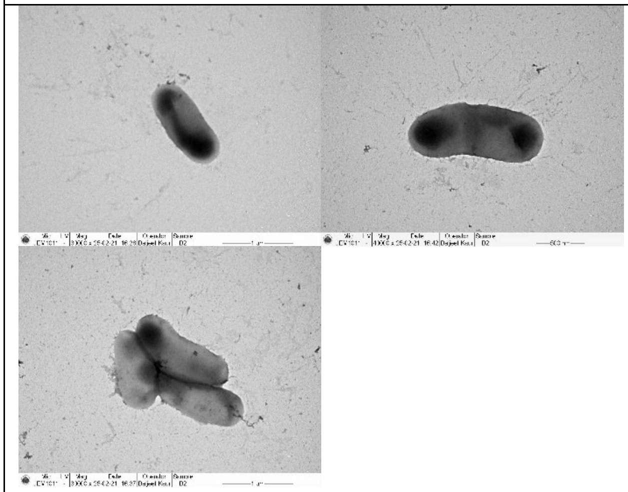

(B) TEM images of *Microbacterium testaceum* B2

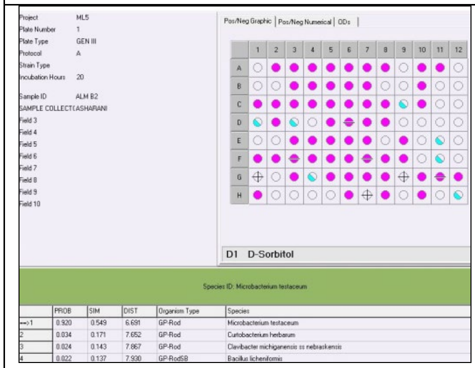

(C) Phenotypic fingerprinting of *Microbacterium testaceum* B2

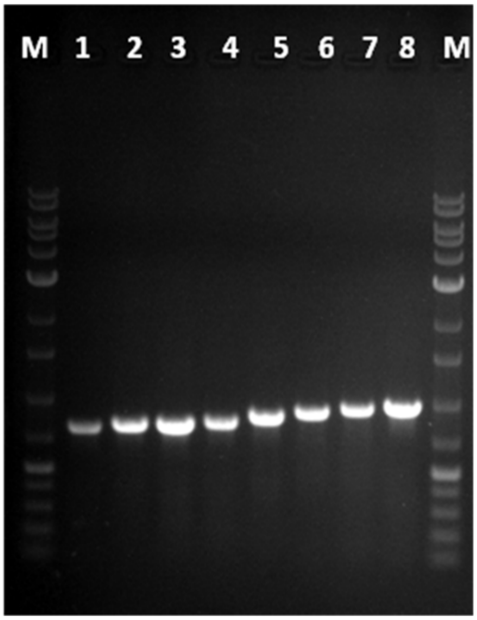

(D) Multigene amplicon profile of *Microbacterium testaceum* B2

Lane M: 1 kb ladder
Lane 1: tyrS (767 bp)
Lane 2: metG (793 bp)
Lane 3: gyrB (795 bp)
Lane 4: cycS (810 bp)
Lane 5: pyk (858 bp)
Lane 6: infB (900 bp)
Lane 7: fumC (929 bp)
Lane 8: rpoC (959 bp)
Lane M: 1 kb ladder

Figure S1. Characterization and identification of endophytic *Microbacterium testaceum* B2.

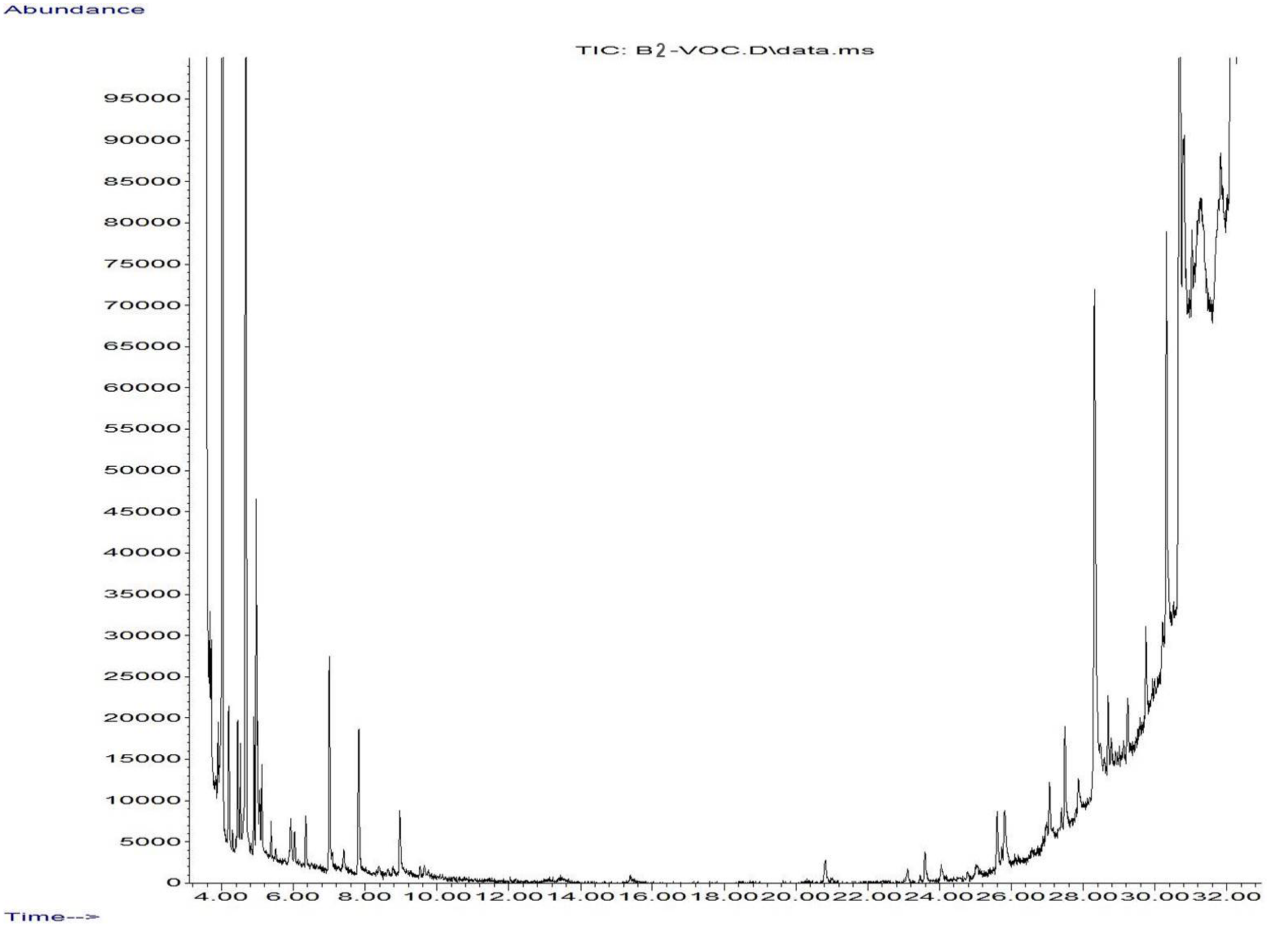

Figure S2. GC-MS chromatogram profile of *Microbacterium testaceum* B2.

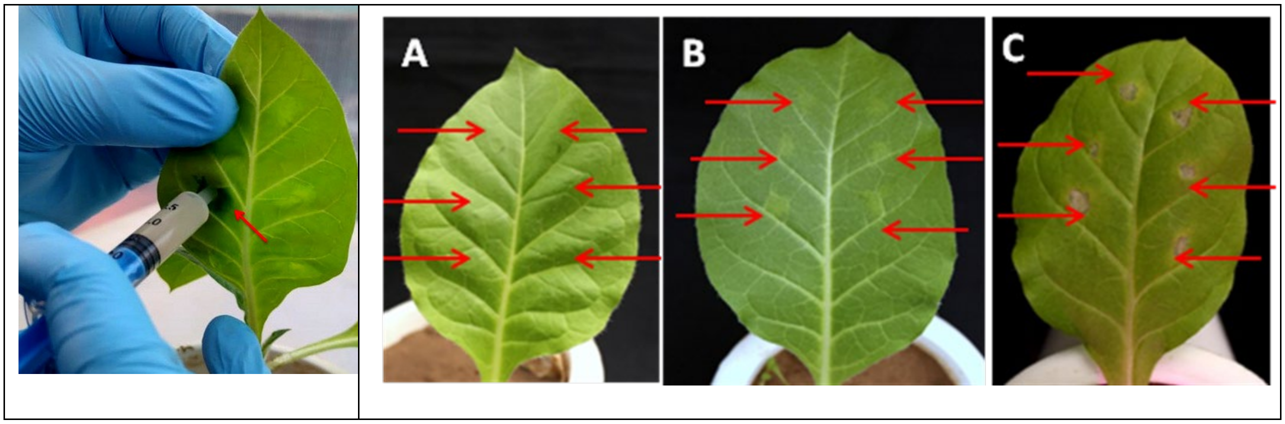

Figure S3. Assay for pathogenic ability of *M. testaceum* B2R by hypersensitivity assay on *N. tabacum* by leaf infiltration. (A) Leaf infiltrated with sterile distilled water (Negative control). (B) Leaf infiltrated with *M. testaceum* B2R. (C) Leaf Infiltrated with *Ralstonia solanacearum* (Positive control).

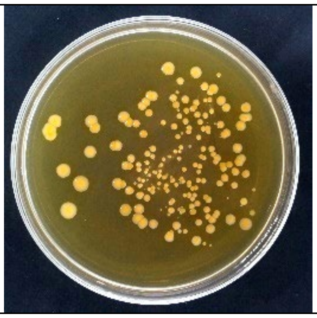	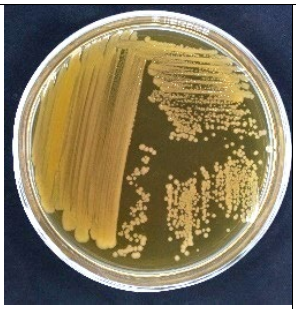	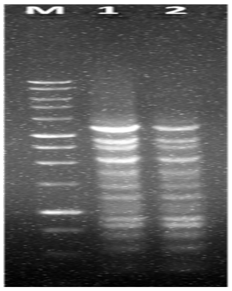 Lane M: 1 kb ladder;<br>Lane 1: *Microbacterium testaceum* B2 (wild type);<br>Lane 2: *Microbacterium testaceum* B2 (rifamycin resistant type)
**A. Selection of Rifamycin resistant type**	**B. Confirmation of Rifamycin resistant type**	**C. BOX-PCR-Based confirmation of genetic purity of rifamycin resistant types**

Figure S4. Selection of rifamycin-resistant *Microbacterium testaceum* B2.

<table>
<tr><td>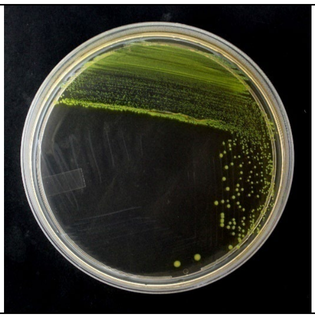</td><td>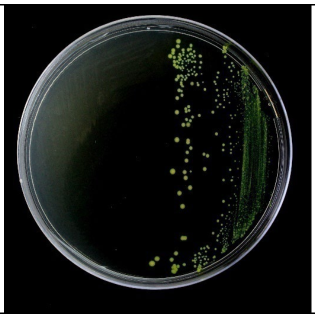</td><td rowspan="3">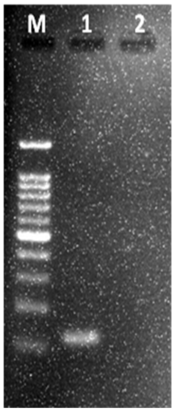
PCR based detection using gfp- specific PCR markers
Lane M: 1 kb ladder; Lane 1: Microbacterium testaceum B2::gfp; Lane 2: Microbacterium testaceum B2</td></tr>
<tr><td>Microbacterium testaceum B2::gfp on NA (Rif50+Gm20)</td><td>Microbacterium testaceum B2::gfp on NA</td></tr>
<tr><td>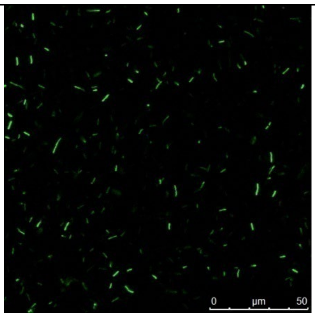</td><td>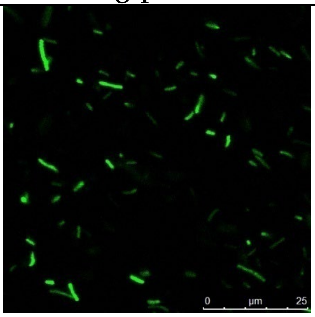</td></tr>
<tr><td>Microbacterium testaceum B2::gfp</td><td>Microbacterium testaceum B2::gfp</td><td></td></tr>
</table>

Figure S5. Genetic transformation of *M. testaceum* B2 for gfp expression.

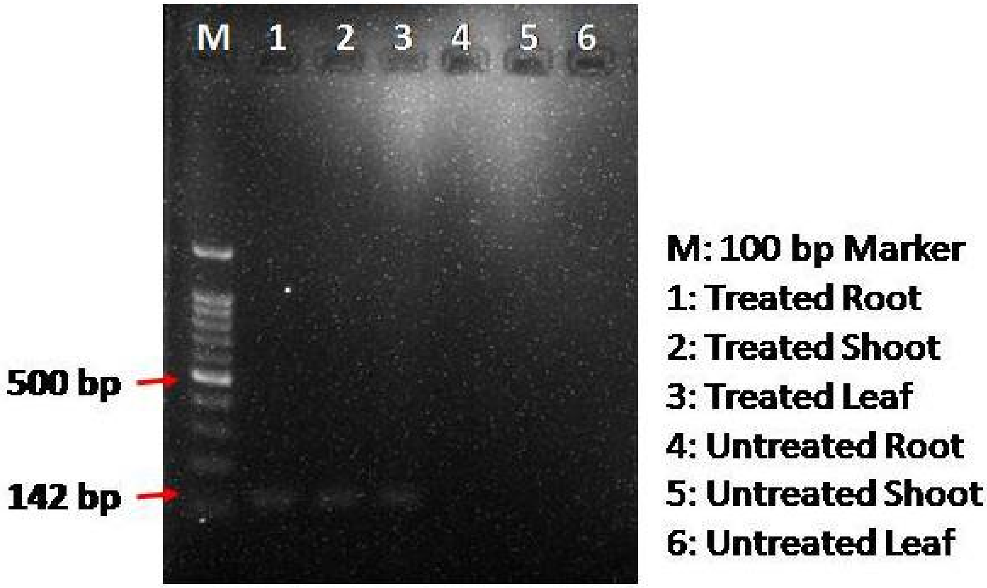

Figure S6. Agarose gel image showing the PCR amplicon of gfp gene inserted in *M. testaceum*

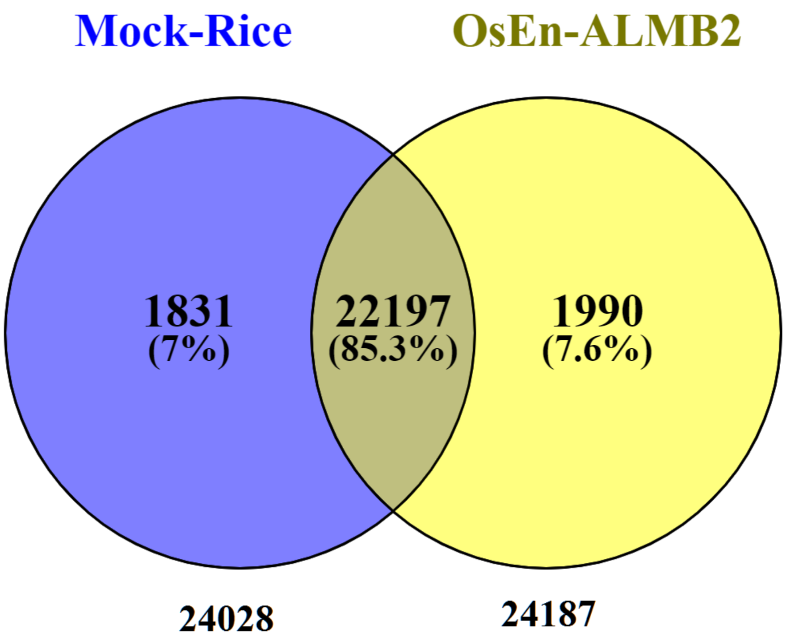

Figure S7. Total number of genes obtained in Mock-Rice and OsEn-ALM_B2 treated rice.

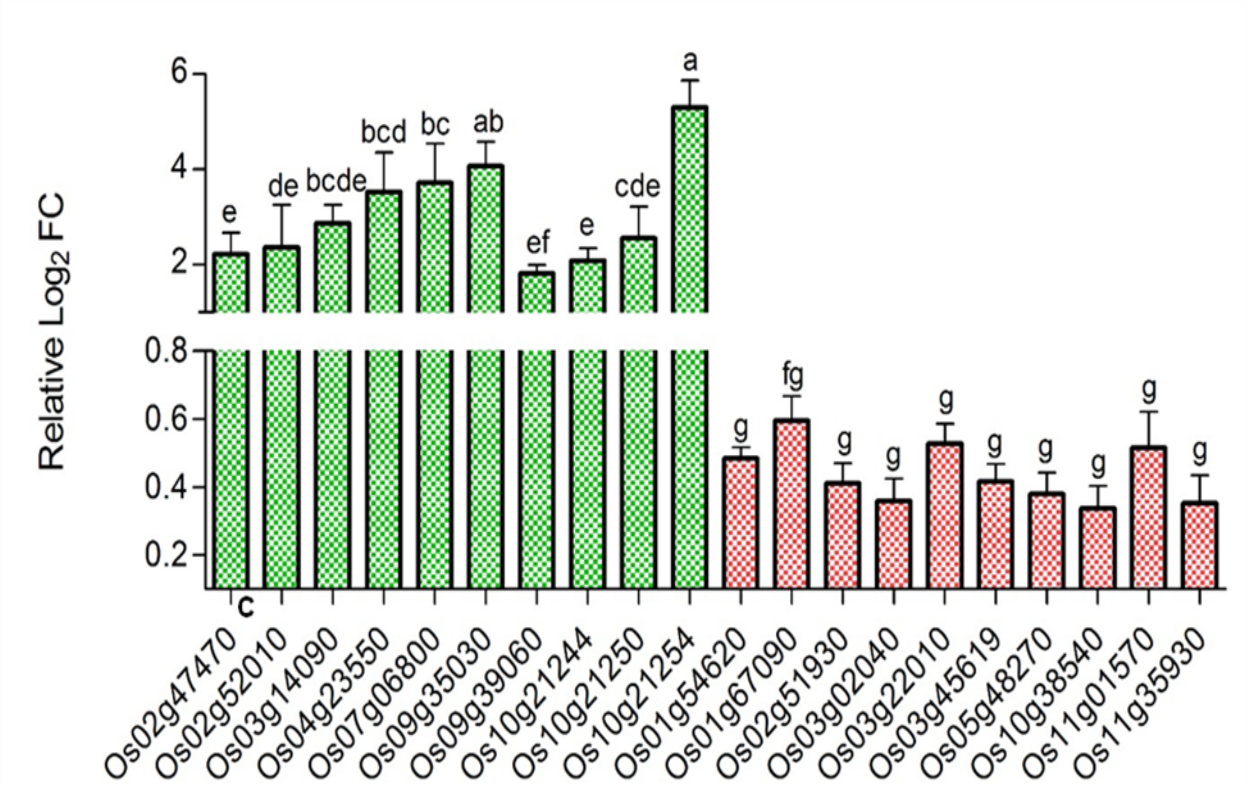

Figure S8. Relative fold change of the up- and down regulated genes in rice upon colonization by *Microbacterium testaceum* OsEnb_ALM_B2. The columns represent the means of biological replicates ± SE. The overall significant differences between treatments are represented by $p \leq 0.001$ (***) as per Tukey's post hoc HSD analysis. The data designated with the same alphabet were significantly similar whereas the data with various alphabets were significantly different from each other. Treatments were found significant at 5% level of significance and critical difference 0.05.

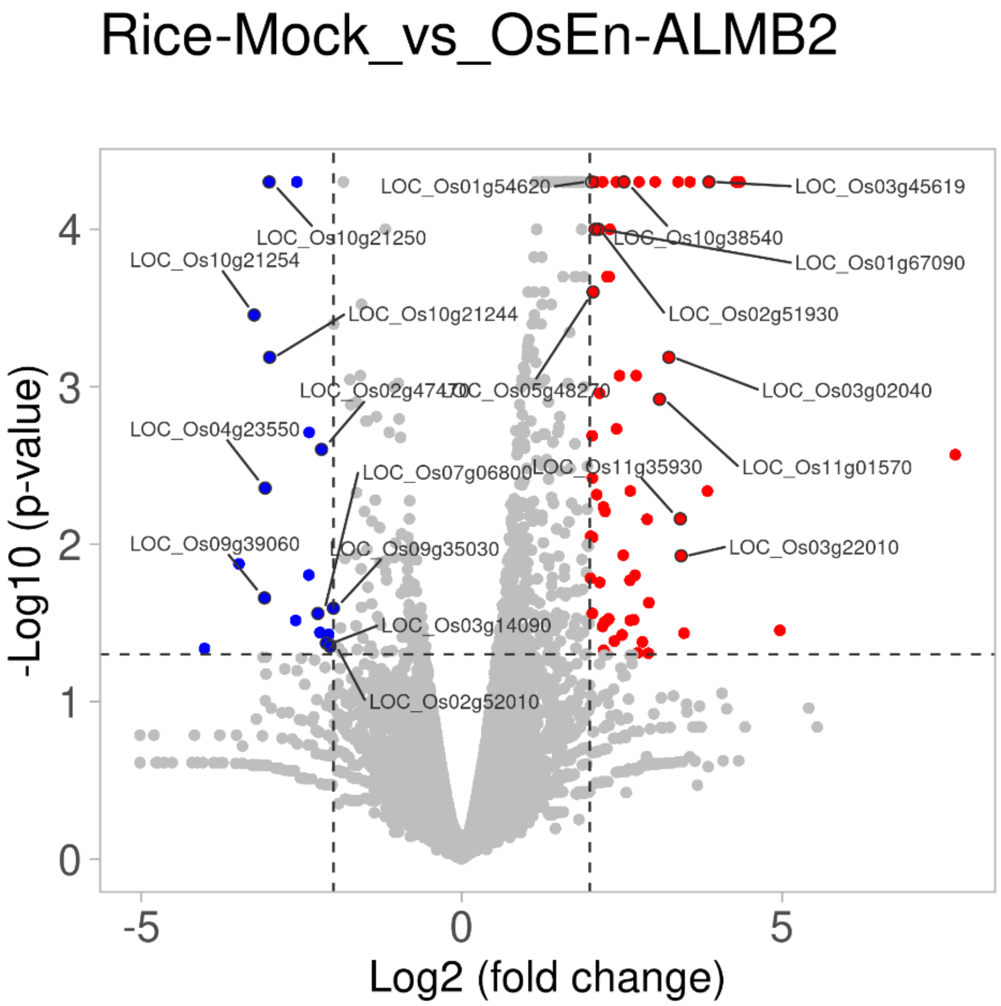

Figure S9. Differential genes expression volcano plot mapped with Log2 fold change expression and –Log10 *p* value. The *p* value cut-off were <0.05 were considered for the significant change.

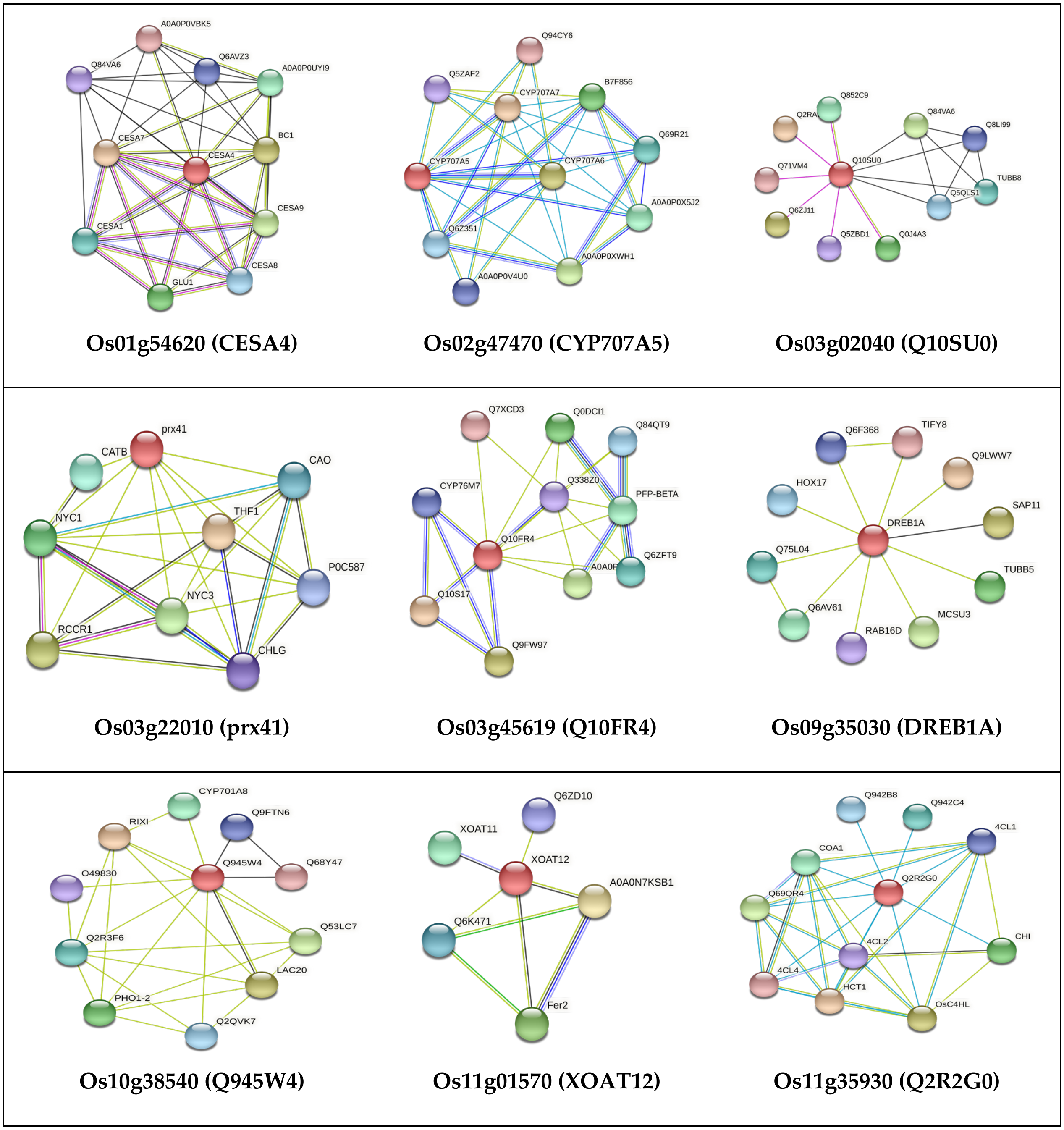

Figure S10. Coexpression analysis of DEGs through String network.
