# Supplementary material for: New Insights on Endophytic Microbacterium-Assisted Blast Disease Suppression and Growth Promotion in Rice: Revelation by Polyphasic Functional Characterization and Transcriptomics
Source: Microorganisms. 2023 Jan 31;11(2):362. doi: 10.3390/microorganisms11020362 (PMC9963279; doi:10.3390/microorganisms11020362)
Supplement: Supplementary file 1 [file microorganisms-11-00362-s001.zip › microorganisms-2043028-Supplementary Tables.pdf]

Table S1. Details of primers used in the study for genetic characterization of *Microbacterium testaceum* D18.

Genes	Gene details and function	Primer ID	Nucleotide sequence (5'-3')	T_m (°C)	Ta Opt(°C)	Amplicon size (bp)
tyrS	Tyrosine--tRNA ligase	MT_tyrS_F<br>MT_tyrS_R	TCTGGGACGAGCTGGTCTAT<br>GTCGGTGTTGAGCCAGAACT	59.8<br>60.3	55	767
metG	Methionine-tRNA ligase	MT_metG_F<br>MT_metG_R	CTTCAAGCTCAGCGAGTTCC<br>CTCGTTGTCGGTGATGTAGC	60.3<br>59.3	55	793
gyrB	DNA gyrase subunit B	MT_gyrB_F<br>MT_gyrB_R	GTGTCTTCGGACTCGACCTC<br>GAAGGTTCCCAACGAGTACG	59.8<br>59.6	55	795
cysS	Cytochrome c, somatic	MT_cysS_F<br>MT_cysS_R	TGACTGTTCGGCTGTACGAC<br>GCGACTTCGACATCTTCTGC	59.9<br>61.1	54	810
pyk	Pyruvate kinase	MT_pyk_F<br>MT_pyk_R	GAACAACAAGGGCATCAACC<br>TCGTTCGACAGCAGGTAGTC	60.4<br>59.0	55	858
infB	Eukaryotic initiation factor IF-2	MT_infB_F<br>MT_infB_R	CAGCTCACCGAGTACGGTCT<br>GAACTTCGAGGAGCGGAACA	60.5<br>60.0	55	900
fumC	Fumarate hydratase	MT_fumC_F<br>MT_fumC_R	TGAACATGAACGAGGTGCTC<br>CTCGTAGCCGATGACCTTGT	59.8<br>60.3	54	929
rpoC	DNA-directed RNA polymerase subunit beta'	MT_rpoC_F<br>MT_rpoC_R	CAGTACTTCGAGGCCCACAT<br>GACGGCTTCAGGATGTTGTT	60.1<br>60.1	55	959

Primers were designed using Primer3 Plus https://www.bioinformatics.nl/cgi-bin/primer3plus/primer3plus.cgi
Primer Tm values and other integrity parameter were tested in http://biotools.nubic.northwestern.edu/OligoCalc.html

Table S2. PCR reaction mixture for amplification of *M. testaceum* specific genes.

Component	Stock concentration	Working concentration	50 µL reaction
Promega PCR Buffer	5 X	1 X	10.0 µL
$MgCl_2$	25.0 mM	1.5 mM	3.0 µL
dNTPs	10.0 mM	200.0 µM	1.0 µL
Taq polymerase	5.0 U μL^{-1}	1.0 U	0.2 µL
Forward primer	10.0 µM	0.2 µM	1.0 µL
Reverse primer	10.0 µM	0.2 µM	1.0 µL
Genomic DNA	100.0 ng μL^{-1}	100.0 ng	1.0 µL
Water	1 X	1 X	32.8µL

Table S3. PCR temperature condition for amplification of *M. testaceum* specific genes.

Steps	Temperature (°C)	Time	
Pre- denaturation	95.0	5.0 minutes	
Cycle-denaturation	94.0	1.0 minutes	35 cycles
Annealing	*	1.0 minutes	
Extension	72.0	1:0 minutes	
Final extension	72.0	5.0 minutes	
Storing	4.0	∞	

* As per the Table S1.

Table S4. Rice defense genes used for the qPCR analysis and their function.

Gene	Characteristics and function	References
*OsFMO1** (Flavin-dependent Monooxygenase 1)	An essential component for induced systemic acquired resistance (SAR)	[43,44]
*PAD4** (Phytoalexin deficient 4)	Phytoalexin deficient 4 (PAD4) induces JA-dependent induced systemic resistance. It also plays an important role in the accumulation of JA and a terpenoid-type phytoalexin mamilactone A (MOA).	[40]
*OsNPR1** (Non-Repressor of Pathogenesis related Protein)	A central regulator of salicylic acid (SA)-mediated defense signaling. Reallocation of energy and resources during defense responses	[42]
*OsEDS1** (Enhanced disease susceptibility 1)	Enhanced disease susceptibility 1 (EDS1) induces JA-dependent induced systemic resistance.	[41]
*OsCERK1*** (Chitin Elicitor Receptor Kinase)	It is a rice receptor-like kinase (RLK) that mediates the signal of a fungal cell wall component chitin. It is indispensable for chitin perception and participates in innate immunity. It can mediate the signaling pathways of both fungal and bacterial PAMP molecules.	[39]
*OsCEBiP*** (Chitin Elicitor Binding Protein)	It is a Pattern Recognition Receptor (PRR) that detects the pathogen PAMP molecule chitin and activates the plant defense system. It is a plasma membrane protein that forms a receptor complex essential for fungal chitin-driven immune responses in rice.	[38]
*Os*Actin	Housekeeping reference gene used in the analysis	

Table S5. List of the PCR primers used in the gene expression studies.

Gene		Primer sequence (5'→3')	Number of bases	Product size (bp)
**OsFMO1*	Forward	CAGTGGAGTGCCCAACATACC	21	65
	Reverse	CCTGGCCATCAAATGCTTCT	20	
**OsPAD4*	Forward	TGCCGACTACCACCGAAAC	19	61
	Reverse	CCGGCCATGGGTGATGTA	18	
**OsNPR1*	Forward	AAACAAAGGAGCAGCTGTATCACA	24	66
	Reverse	CTCCGGCAGATACTCATTGCA	21	
**OsEDS1*	Forward	TTGAATTTTGTCGTGCCAGTAGA	23	63
	Reverse	GGCAGATGCAAGCGGAGTAA	20	
***OsCERK1*	Forward	AAGAACTACCGGGCAAAGGT	20	244
	Reverse	GCCCCTTTGAATCACTTGAA	20	
***OsCEBiP*	Forward	GTGCGGAGAAGTCTGGAAAG	20	131
	Reverse	TCCTGATTTCGCTTGCTTTT	20	
**OsActin*	Forward	CAGCCACACTGTCCCCATCTA	21	67
	Reverse	AGCAAGGTCGAGACGAAGGA	20	

*[16,20]

**Primers designed using online Platform Primer3Plus http://www.bioinformatics.nl/cgi-bin/primer3plus/primer3plus.cgi

Table S6. Reaction condition for one step qRT-PCR reaction.

**Steps**	**Temperature (°C)**	**Time**	
Reverse transcription	37.0	15 minutes	1 cycle
Reverse-transcriptase inactivation	95.0	10 minutes	
Denaturation	95.0	10 seconds	30 cycles
Annealing	58.0	30 seconds	
Extension	72.0	30 seconds	
Melting	95.0	10 seconds	1 cycle
Melting	63.0	60 seconds	
Melting	97.0	1 second	
Final cooling	37.0	30 seconds	

Table S7. Primers used for validation of transcriptome data.

Gene name	F/R	Primer	BASE	Tm	Product size
Os02g47470	F	CAAGGGCTGGAAAGTGCTAC	20	59.9	211
	R	GTACTTGGTTGCGAGGTGGT	20	60.0	
Os02g52010	F	GCAACTCACGTACCACAACG	20	59.8	191
	R	TGGACAGGTACAGCTGGTTG	20	59.6	
Os03g14090	F	GAACCATCCAGGAGCACAGT	20	60.1	201
	R	CCGATTGCATCTGGTTCTTT	20	60.1	
Os04g23550	F	GACGCAGGATGACTCCAACT	20	60.3	201
	R	CGCTCCATGAGGATGTTCTT	20	60.2	
Os07g06800	F	GGCAACTTCTACCACCACTAC	21	62.0	273
	R	GCTATCAGGGAAATCCTCGAAC	22	62.0	
Os09g35030	F	GACCAAGTTCAGGGAGACGA	17	61.0	210
	R	GAGTCGGCGAAGTTGAGG	17	61.0	
Os09g39060	F	GTATTTCCCGGTCCTGCAT	19	62.0	234
	R	TTGTAGAGCTGGAGGCTTTG	20	62.0	
Os10g21244	F	TTCAGAGAGAGAGGAAGCGG	20	58.5	244
	R	TCTCGAAGTATGTGTCCGGA	20	57.9	
Os10g21250	F	TAGTTTGGCCAATCGTAGGG	20	60.0	227
	R	TTGGAACCAGGCTAATTTGG	20	59.9	
Os10g21254	F	TATTTCCGGGCACTAGAACG	20	60.1	213
	R	GCGCTTCGTAATCTTCAACC	20	59.9	
Os01g54620	F	CCTGAGGAAGGCTGGATTATG	21	62.0	174
	R	GTGTTGGTACCCTGGTCTTT	20	62.0	
Os01g67090	F	CCCTAAGGACAAGCCTACAAA	21	62.0	173
	R	GGTACAGCGGGATGTTGAT	19	62.0	
Os02g51930	F	CCCCACTCAAAGATGTCGAT	20	59.9	202
	R	CCGAAGGAGTTGACGATGAT	20	61.1	
Os03g02040	F	CTGGGAGGAAAGCGAGAAAG	22	58.3	176
	R	ATGCCAGCTTGTTCTTCAGC	18	59.1	
Os03g22010	F	CCTTCTTCACCGACCTCATC	20	59.7	220
	R	CAGTTGACCCTGGAGCAGTT	20	60.3	
Os03g45619	F	GTGCTGGTACCCTGATTCGT	20	60.0	144
	R	CTTGGCTGTGACGCTGAATA	20	60.0	
Os05g48270	F	TCCTCGTCTCCAACTCCAAC	20	60.2	199
	R	CCAGTGGTGAAATCGAAGGT	20	60.0	
Os10g38540	F	TCATCGTGCAGTACCTCGAC	20	59.9	235
	R	CCCTTGGAACACTCCTTGAA	20	60.1	
Os11g01570	F	TTACTCCGGCTTGAAGTGCT	20	60.0	183
	R	ACTGAATCGCCCACAAAAAC	20	60.0	
Os11g35930	F	AAGAGCACGTTCCACGAGAT	20	59.4	162
	R	GCCATACTGCCTCATCACCT	20	59.5	

Table S8. List of bacterial species used in molecular phylogenetic analysis.

Isolates/Strains	Gen Bank Accession	Range in genome	Habitat/Source of isolation	Placement of bacterial isolate with more than 50% Boostrap support
**Gene- tyrS (727 bp)**				
**OsEnb_ALM_B2**			Rice endophyte: This study	OsEnb_ALM_B2 (gene- tyrS) with *Microbacterium testaceum* strain StLB037
Microbacterium testaceum strain StLB037	AP012052.1	c266577-265818	Potato leaves endophyte	
Microbacterium oryzae strain MB-10	CP032550.1	c1299862-1299103	Rice field soil	
Microbacterium wangchenii strain dk512	CP038266.1	1357863-1358622	Tibetan Gazelle	
Microbacterium resistens strain MZT7	CP082781.1	3516938-3517690	Activated sludge	
Microbacterium sediminis strain YLB-01	CP038256.1	c1934828-1934074	Deep-sea sediment	
Microbacterium esteraromaticum strain B24	CP043732.1	c3812-3053	Agricultural soil	
Microbacterium caowuchunii strain ST-M6	CP044231.1	c2075093-2074335	Ochotona curzoniae	
Microbacterium oxydans strain HG3	CP031422.1	1383966-1384699	Radionuclide-contaminated soil	
**Gene-metG (784 bp)**				
**OsEnb_ALM_B2**			Rice endophyte: This study	OsEnb_ALM_B2 (gene- metG) with *Microbacterium testaceum* strain StLB037
Microbacterium testaceum strain StLB037	AP012052.1	3194351-3195134	Potato leaves endophyte	
Microbacterium liquefaciens strain DND4	CP101471.1	c2591322-2590539	Poultry farm	
Microbacterium oxydans strain VIU2A	CP031338.1	c2685627-2684844	Radionuclide contaminated soil	
Microbacterium paraoxydans strain DSM15019	LT629770.1	c1031240-1030457	Child's blood	
Microbacterium paludicola strain CC3	CP018134.1	345670-346453	Farmland soil	
Microbacterium wangchenii strain dk512	CP038266.1	c2550399-2549616	Tibetan Gazelle	
Microbacterium lushaniae strain L-031	CP044232.1	c2445112-2444329	*Ochotona curzoniae*	
Microbacterium hominis strain PA2F3	CP054038.1	1415975-1416758	Intestine of *Perinereis linea*	
**Gene- gyrB (764 bp)**				
**OsEnb_ALM_B2**			Rice endophyte: This study	OsEnb_ALM_B2 (gene- gyrB) with *Microbacterium testaceum* strain StLB037
Microbacterium testaceum strain StLB037	AP012052.1	1598792-1599576	Potato leaves endophyte	
Microbacterium hominis strain PDNC016	CP070346.1	1903532-1904313	Plastic debris in land/lake	
Microbacterium oleivorans strain I46	CP058316.1	1933915-1934696	Soil	
Microbacterium paludicola strain CC3	CP018134.1	2330718-2331507	Farmland soil	
Microbacterium chocolatum strain SIT 101	CP015810.1	c792461-791671	Soil	
Microbacterium lemovicicum strain Viu22	CP031423.1	c5858-5074	Natural uranium-rich soil	
Microbacterium caowuchunii strain ST-M6	CP044231.1	c5698-4920	*Ochotona curzoniae*	
Microbacterium wangchenii strain dk512	CP038266.1	c5633-4856	Tibetan Gazelle	
**Gene- cycS (770 bp)**				
**OsEnb_ALM_B2**			Rice endophyte: This study	OsEnb_ALM_B2 (gene- cycS) with *Microbacterium testaceum* strain StLB037
Microbacterium testaceum strain StLB037	AP012052.1	2968120-2968919	Potato leaves endophyte	
Microbacterium pygmaeum strain DSM 23142	LT629692.1	1770947-1771757	Soil	
Microbacterium caowuchunii strain ST-M6	CP044231.1	865282-866089	*Ochotona curzoniae*	
Microbacterium paraoxydans strain 70447	CP064873.1	c2516563-2515785	Human	
Microbacterium sediminis strain YLB-01	CP038256.1	c2147966-2147168	Deep-sea sediment	
Microbacterium oryzae strain MB-10	CP032550.1	2893694-2894492	Rice field in India	
Microbacterium wangchenii strain dk512	CP038266.1	c2730954-2730147	Tibetan Gazelle	
Microbacterium protaetiae strain DFW100M-13	CP035494.1	c264377-263579	*Protaetiabrevitarsis seulensis* larva	
**Gene- pyk (836 bp)**				
**OsEnb_ALM_B2**			Rice endophyte: This study	OsEnb_ALM_B2 (gene- pyk) with *Microbacterium testaceum* strain StLB037
Microbacterium testaceum strain StLB037	AP012052.1	857364-3858205	Potato leaves endophyte	
Microbacterium hominis strain PA2F3	CP054038.1	2055565-2056406	Intestinal content	
Microbacterium lemovicicum strain Viu22	CP031423.1	c1434715-1433876	Natural uranium-rich soil	
Microbacterium foliorum strain 122	CP019892.1	c1836827-1835986	Plant	
Microbacterium caowuchunii strain ST-M6	CP044231.1	c1623811-1622974	*Ochotona curzoniae*	
Microbacterium binotii strain Au-Mic3	CP090347.1	c1686237-1685402	Soil	
Microbacterium pygmaeum strain DSM 23142	LT629692.1	2703815-2704652	Soil	
Microbacterium lushaniae strain L-031	CP044232.1	c1740101-1739260	*Ochotona curzoniae*	
**Gene- infB (722 bp)**				
**OsEnb_ALM_B2**			Rice endophyte: This study	OsEnb_ALM_B2 (gene- infB) with *Microbacterium*
Microbacterium testaceum strain StLB037	AP012052.1	c376684-375798	Potato leaves endophyte	
Microbacterium hominis strain PA2F3	CP054038.1	c2448488-2447602	Intestinal content	

Microbacterium oleivorans strain A9	CP031421.1	c2077267-2076384	Radionuclide contaminated soil	*testaceum* strain StLB037
Microbacterium chocolatum strain SIT 101	CP015810.1	791671-792461	Soil	
Microbacterium lushaniae strain L-031	CP044232.1	4890-5667	*Ochotona curzoniae*	
Microbacterium binotii strain Au-Mic3	CP090347.1	c2257349-2256463	Soil	
Microbacterium paludicola strain CC3	CP018134.1	2330718-2331507	Farmland soil	
Microbacterium hydrocarbonoxydans strain Marseille-P2596	LR882982.1	1365932-1366818	Permafrost	
**Gene- fumC (899 bp)**				
**OsEnb_ALM_B2**			Rice endophyte: This study	OsEnb_ALM_B2 (gene- fumC) with *Microbacterium testaceum* strain StLB037
Microbacterium testaceum strain StLB037	AP012052.1	c3271555-3270648	Potato leaves endophyte	
Microbacterium aurum strain KACC 15219	CP018762.1	c1435731-1434824	Corn steep liquor	
Microbacterium lemovicicum strain Viu22	CP031423.1	2041092-2041996	Natural uranium-rich soil	
Microbacterium binotii strain Au-Mic3	CP090347.1	c1193314-1192407	Soil	
Microbacterium resistens strain MZT7	CP082781.1	c217998-217091	Activated sludge	
Microbacterium oleivorans strain A9	CP031421.1	1777862-1778769	Radionuclide-contaminated soil	
Microbacterium paraoxydans strain 70447	CP064873.1	c1103522-1102621	Human	
Microbacterium paludicola strain CC3	CP018134.1	c427371-426464	Farmland soil	
**Gene- rpoC (932 bp)**				
**OsEnb_ALM_B2**			Rice endophyte: This study	OsEnb_ALM_B2 (gene- rpoC) with *Microbacterium testaceum* strain StLB037
Microbacterium testaceum strain StLB037	AP012052.1	c1019750-1018816	Potato leaves endophyte	
Microbacterium chocolatum strain SIT 101	CP015810.1	1178231-1179165	Soil	
Microbacterium esteraromaticum strain B24	CP043732.1	1913627-1914559	Agricultural soil	
Microbacterium paraoxydans strain 70447	CP064873.1	782000-782934	Human	
Microbacterium hominis strain PDNC016	CP070346.1	c1496987-1496056	Plastic debris in land/lake	
Microbacterium oxydans strain VIU2A	CP031338.1	971383-972317	Radionuclide-contaminated soil	
Microbacterium oleivorans strain I46	CP058316.1	2230650-2231584	Soil	
Microbacterium foliorum strain NRRL B-24224	CP031425.1	878106-879040	Phyllosphere of grasses	

All the strain used in the Phylogenetic study are from whole genome sequence

Evolutionary analysis by Maximum Likelihood method

The evolutionary history was inferred by using the Maximum Likelihood method and Hasegawa-Kishino-Yano model [27]. The tree with the highest log likelihood (-3465.91) is shown. The percentage of trees in which the associated taxa clustered together is shown above the branches. Initial tree(s) for the heuristic search were obtained automatically by applying Neighbor-Join and BioNJ algorithms to a matrix of pairwise distances estimated using the Maximum Composite Likelihood (MCL) approach, and then selecting the topology with superior log likelihood value. This analysis involved 9 nucleotide sequences. Codon positions included were 1st+2nd+3rd+Noncoding. There were a total of 759 positions in the final dataset. Evolutionary analyses were conducted in MEGA11 as reported by Kumar et al. [1]*.

Table S9. Plant probiotic traits of *Microbacterium testaceum* B2.

S. No.	Traits	Reaction
1	Ammomia production	+
2	IAA production	+
3	P solubilization assay	+
4	K solubilization assay	+
5	Zn solubilization assay	+
6	Siderophore assay	+
7	Cellulase Assay	+
8	Chitinase Assay	+
9	Xylanase Assay	−
10	Amylase assay	−
11	Pectinase assay	−
12	Protinase Assay	−

+ Positive for solubilization/activity;

- Negative for solubilization/activity

Table S10. Expression analysis of defense related genes in rice upon bacterization by *Microbacterium testaceum* B2.

Defense genes	Pusa Basmati 1				BPT 5204			
	Seedling bacterization (CFU mL^{-1})		Leaf bacterization (CFU mL^{-1})		Seedling bacterization (CFU mL^{-1})		Leaf bacterization (CFU mL^{-1})	
	10^8	10^7	10^8	10^7	10^8	10^7	10^8	10^7
OsFMO	1.11	1.39	0.75	0.19	0.96	1.32	1.03	1.29
OsPAD4	0.85	0.90	1.94	0.90	0.95	1.04	0.78	1.11
OsNPR1	2.09	2.11	2.18	1.18	1.03	1.19	1.20	1.04
OsEDS1	1.43	1.69	1.79	0.83	1.46	1.01	0.87	0.70
OsCERK1	1.64	1.65	3.66	0.80	2.02	0.82	0.76	1.16
OsCEBiP	0.32	0.96	1.09	2.72	0.35	0.40	0.49	0.16

Table S11. RNA-Seq Data Statistics.

	Rice-Mock 1		Rice-Mock 2		OsEn-ALMB2 -1		OsEn-ALMB2 -2	
	**R1**	**R2**	**R1**	**R2**	**R1**	**R2**	**R1**	**R2**
**Number of raw reads**	56,212,177	56,212,177	35,589,139	35,589,139	33,873,973	33,873,973	33,492,401	33,492,401
**Number of bases (MB)**	8,431.83	8,431.83	5,338.37	5,338.37	5,081.10	5,081.10	5,023.86	5,023.86
**Mean Phred score (Q)**	36.26	35.74	36.28	35.27	36.27	35.16	36.35	35.71
**GC (%)**	49.87	49.86	45.02	45.56	45.64	46.25	44.98	45.48
**%data >=Q30**	95.72	93.33	95.70	91.11	95.62	90.75	96.07	92.97
**Raw read length (bp)**	150.00	150.00	150.00	150.00	150	150	150	150
**Total Read Count**	112,424,354		71,178,278		67,747,946		66,984,802	
**Read Count after rRNA removal**	70,844,732		70,656,064		67,257,628		66,746,966	
**QC Pass %**	63.02		99.27		99.28		99.64	
**Aligned Read Count**	62,387,306		62,691,039.00		58,561,996		59,214,817	
**Aligned %**	88.06		88.73		87.07		88.72	
**Unaligned %**	11.94		11.27		12.93		11.28	

Table S12 List of up-regulated DEGs

Gene_id	Gene	log2 (fold change)	Putative Function
XLOC_000032	LOC_Os01g01430	2.8194	No apical meristem protein, putative, expressed
XLOC_004048	LOC_Os01g16030	2.46233	ADP-ribosylation factor, putative, expressed
XLOC_004291	LOC_Os01g21970	2.04181	Protein kinase, putative, expressed
XLOC_001899	LOC_Os01g44130	2.74881	Pspartic proteinase oryzasin-1 precursor, putative, expressed
XLOC_001940	LOC_Os01g45460	2.29496	Serine esterase, putative, expressed
XLOC_002330	LOC_Os01g53240	2.01618	BURP domain-containing protein, expressed
XLOC_002405	LOC_Os01g54620	2.02863	CESA4 - cellulose synthase, expressed
XLOC_006116	LOC_Os01g65690	2.3014	4,5-DOPA dioxygenase extradiol, putative, expressed
XLOC_003006	LOC_Os01g67090	2.13654	IQ calmodulin-binding motif domain-containing protein, expressed
XLOC_019172	LOC_Os02g21890	2.22597	Expressed protein
XLOC_022301	LOC_Os02g33550	3.46785	Harpin-induced protein 1 domain-containing protein, expressed
XLOC_019971	LOC_Os02g40100	2.76844	Plant protein of unknown function DUF869 domain-containing protein, expressed
XLOC_020024	LOC_Os02g41780	2.72275	Transporter-related, putative, expressed
XLOC_020260	LOC_Os02g46830	2.62934	Expressed protein
XLOC_020525	LOC_Os02g51930	2.08085	Cytokinin-O-glucosyltransferase 2, putative, expressed
XLOC_026399	LOC_Os03g02040	3.233	Remorin, putative, expressed
XLOC_023631	LOC_Os03g02660	2.91282	Expressed protein
XLOC_026873	LOC_Os03g13200	4.96497	Peroxidase precursor, putative, expressed
XLOC_027147	LOC_Os03g18980	2.15221	Kinesin motor domain-containing protein, expressed
XLOC_024567	LOC_Os03g22010	3.42439	Peroxidase precursor, putative, expressed
XLOC_024859	LOC_Os03g29150	3.83451	NAD-dependent epimerase/dehydratase family protein, putative, expressed
XLOC_028251	LOC_Os03g45619	3.85804	Cytochrome P450, putative, expressed
XLOC_028782	LOC_Os03g57460	2.41659	Fasciclin domain-containing protein, expressed
XLOC_026071	LOC_Os03g58490	2.15413	DUF593 domain-containing protein, expressed
XLOC_026343	LOC_Os03g64230	2.91992	Expressed protein
XLOC_031719	LOC_Os04g01710	2.21118	Cysteine proteinase At4g11310 precursor, putative, expressed
XLOC_030289	LOC_Os04g28620	7.70157	Male sterility protein, putative, expressed
XLOC_033174	LOC_Os04g33450	2.54289	Expressed protein

XLOC_031427	LOC_Os04g53800	2.2369	Leucoanthocyanidin reductase, putative, expressed
XLOC_031530	LOC_Os04g56060	2.03968	Protein kinase domain-containing protein, expressed
XLOC_034587	LOC_Os05g04500	2.09977	Peroxidase precursor, putative, expressed
XLOC_037108	LOC_Os05g09740	4.34003	HAD superfamily phosphatase, putative, expressed
XLOC_035071	LOC_Os05g15880	2.62881	Glycosyl hydrolase, putative, expressed
XLOC_038353	LOC_Os05g38230	2.31284	Oxidoreductase, aldo/keto reductase family protein, putative, expressed
XLOC_038654	LOC_Os05g45140	2.50216	Glucosyl transferase, putative, expressed
XLOC_038657	LOC_Os05g45180	2.20008	Anthocyanidin 5,3-O-glucosyltransferase, putative, expressed
XLOC_036507	LOC_Os05g48270	2.05005	Auxin-responsive protein, putative, expressed
XLOC_039211	LOC_Os06g05980	2.01058	Transporter family protein, putative, expressed
XLOC_039484	LOC_Os06g11490	2.03542	Plastocyanin-like domain-containing protein, putative, expressed
XLOC_039904	LOC_Os06g21360	2.38126	Transporter, monovalent cation:proton antiporter-2 family, putative, expressed
XLOC_039956	LOC_Os06g22330	2.03837	Expressed protein
XLOC_042653	LOC_Os06g30950	2.09231	Transporter-related, putative, expressed
XLOC_043076	LOC_Os06g39390	2.62491	Transferase family protein, putative, expressed
XLOC_043703	LOC_Os07g01600	2.70388	Dirigent, putative, expressed
XLOC_047927	LOC_Os07g45060	2.07057	Uncharacterized GPI-anchored protein At5g19240 precursor, putative, expressed
XLOC_051380	LOC_Os08g25839	2.5215	Expressed protein
XLOC_051678	LOC_Os08g32160	2.89294	Oxidoreductase, 2OG-FeII oxygenase domain-containing protein, putative, expressed
XLOC_049815	LOC_Os08g36760	3.01985	Remorin C-terminal domain-containing protein, putative, expressed
XLOC_049880	LOC_Os08g38170	2.41425	Methyladenine glycosylase, putative, expressed
XLOC_050177	LOC_Os08g44250	2.19802	Fiber protein Fb14, putative, expressed
XLOC_054086	LOC_Os09g03140	2.0114	Cytokinin-O-glucosyltransferase 2, putative, expressed
XLOC_052841	LOC_Os09g13650	3.37796	Microtubule-associated protein, putative, expressed
XLOC_053553	LOC_Os09g29710	2.26527	Beta-expansin precursor, putative, expressed
XLOC_007765	LOC_Os10g32810	2.26774	Beta-amylase, putative, expressed
XLOC_008024	LOC_Os10g38540	2.53082	Glutathione S-transferase, putative, expressed
XLOC_010056	LOC_Os11g01570	3.0873	PMR5, putative, expressed
XLOC_010162	LOC_Os11g03820	2.10626	D-mannose binding lectin family protein, expressed
XLOC_011087	LOC_Os11g26190	2.68443	RALFL23 - Rapid ALkalinization Factor RALF family protein precursor, expressed

XLOC_011510	LOC_Os11g35930	3.41111	Chalcone synthase, putative, expressed
XLOC_011829	LOC_Os11g42500	3.56245	Dirigent, putative, expressed
XLOC_011834	LOC_Os11g42550	4.27812	Dirigent, putative, expressed
XLOC_014882	LOC_Os12g15530	2.2159	Retrotransposon protein, putative, unclassified, expressed

Table S13 List of down-regulated DEGs.

Gene_id	Gene	log2 (fold change)	Putative Function
XLOC_020171	LOC_Os02g44880	-2.59119	Expressed protein
XLOC_020294	LOC_Os02g47470	-2.1864	Cytochrome P450, putative, expressed
XLOC_023206	LOC_Os02g52010	-2.05338	Phosphate-induced protein 1 conserved region domain containing protein, expressed
XLOC_026919	LOC_Os03g14090	-2.11073	Armadillo/beta-catenin repeat family protein, putative, expressed
XLOC_029732	LOC_Os04g16775	-3.47343	Conserved hypothetical protein
XLOC_029740	LOC_Os04g16842	-4.01198	DNA-directed RNA polymerase subunit alpha, putative, expressed
XLOC_032734	LOC_Os04g23550	-3.06636	Basic helix-loop-helix family protein, putative, expressed
XLOC_031080	LOC_Os04g45960	-2.38465	OsSub42 - Putative Subtilisin homologue, expressed
XLOC_041444	LOC_Os06g04240	-2.21165	Expressed protein
XLOC_046256	LOC_Os07g06800	-2.24364	3-oxo-5-alpha-steroid 4-dehydrogenase, putative, expressed
XLOC_055465	LOC_Os09g35010	-2.37994	Dehydration-responsive element-binding protein, putative, expressed
XLOC_055467	LOC_Os09g35030	-2.00115	Dehydration-responsive element-binding protein, putative, expressed
XLOC_055643	LOC_Os09g39060	-3.07592	Vignain precursor, putative, expressed
XLOC_007240	LOC_Os10g19970	-2.07325	Expressed protein
XLOC_009064	LOC_Os10g21244	-2.99602	Chloroplast 30S ribosomal protein S14, putative, expressed
XLOC_009065	LOC_Os10g21248	-2.57164	Photosystem I P700 chlorophyll a apoprotein A2, putative, expressed
XLOC_009066	LOC_Os10g21250	-3.00382	Photosystem I P700 chlorophyll a apoprotein A1, putative, expressed
XLOC_009067	LOC_Os10g21254	-3.23738	Photosystem I assembly protein ycf3, putative, expressed

*References (Other than MS file)

1. Kumar, S.; Stecher, G.; Tamura, K. MEGA 11: Molecular Evolutionary Genetics Analysis Version 11. *Mol. Biol. Evol.* **2021,** *38*, 3022–3027. https://doi.org/10.1093/molbev/msab120.
